# Dietary patterns and blood-based biomarkers of Alzheimer's disease in cognitively intact older adults: Findings from a population-based study

**DOI:** 10.1016/j.tjpad.2025.100124

**Published:** 2025-03-14

**Authors:** Anja Mrhar, Adrián Carballo-Casla, Giulia Grande, Martina Valletta, Claudia Fredolini, Laura Fratiglioni, Milica Gregorič Kramberger, Aleš Kuhar, Bengt Winblad, Amaia Calderón-Larrañaga, Davide Liborio Vetrano

**Affiliations:** a Aging Research Center, Department of Neurobiology, Care Sciences and Society, Karolinska Institutet and Stockholm University, Stockholm, Sweden; b Biotechnical Faculty, University of Ljubljana, Ljubljana, Slovenia; c Center for Networked Biomedical Research in Epidemiology and Public Health (CIBERESP), Madrid, Spain; d Stockholm Gerontology Research Center, Stockholm, Sweden; e Department of Protein Science, SciLifeLab, KTH Royal Institute of Technology, Solna, Sweden; f Affinity Proteomics Unit, SciLifeLab, KTH Royal Institute of Technology, Solna, Sweden; g Department of Neurology, University Medical Centre Ljubljana, Ljubljana, Slovenia; h Faculty of Medicine, University of Ljubljana, Ljubljana, Slovenia; i Department of Neurobiology, Care Sciences and Society, Division of Clinical Geriatrics, Karolinska Institutet, Huddinge, Sweden; j Department of Neurobiology, Care Sciences and Society, Division of Neurogeriatrics, Karolinska Institutet, Solna, Sweden; k Theme Inflammation and Aging, Karolinska University Hospital, Huddinge, Sweden

**Keywords:** Neurodegeneration, Total tau, Phosphorylated tau, Amyloid beta, Neurofilament light, Glial fibrillary acidic protein, Diet quality, Mediterranean diet, Cohort study, Dementia, Prevention

## Abstract

**Background:**

Diet can impact cognitive aging, but comprehensive data from human studies is lacking and the underlying biological mechanisms are still not fully understood.

**Objectives:**

To investigate the associations between two dietary patterns consistently linked to inflammation and brain health [the Mediterranean diet (MDS) and inflammatory potential of diet (EDII)] and five blood-based biomarkers of Alzheimer´s disease (AD) in a sample of dementia-free community-dwelling older adults.

**Design and setting:**

We used cross-sectional data from the Swedish National Study on Aging and Care in Kungsholmen (SNAC-K).

**Participants:**

Participants who were institutionalized, had dementia or Parkinson's disease, or had missing data on diet and/or biomarkers were excluded. Our study sample consisted of 1907 adults ≥60 years old.

**Measurements:**

Adherence to the MDS and EDII was assessed using a validated food frequency questionnaire. T-tau, p-tau181, Aβ 42/40, NfL, and GFAP were measured in serum. Associations were estimated through quantile regression models at the 25th, 50th, and 75th percentiles of the biomarkers’ levels, and were adjusted for potential confounders and stratified by sex, age, and *APOE-e4* genotype.

**Results:**

In the whole sample, higher adherence to the MDS was associated with lower levels of p-tau181 at the 50th and 75th percentiles [β (95% CI) per 1-SD increment = -0.028 (-0.053, -0.002) and -0.036 (-0.072, -0.001), respectively], while higher adherence to the EDII was associated with higher levels of NfL at the 75th percentile [β (95% CI) per 1-SD increment =0.031 (0.008, 0.053)]. Associations with other biomarkers were only apparent at lower levels of their distribution. Subgroup analyses showed: 1) a stronger inverse association between the MDS and p-tau181 in APOE-e4 carriers than non-carriers, and 2) an inverse association of the MDS with GFAP only in participants ≥78 years.

**Conclusions:**

Diet seems to be associated with biomarkers of AD pathology in cognitively intact older adults. Some associations were more apparent in the presence of genetic predisposition for AD or advanced age.

## 1 Introduction

Dementia is a rapidly growing public health problem affecting more than 55 million people worldwide, a figure that is expected to further increase. Alzheimer's disease (AD) is the most common form of dementia, representing 60 to 70 % of cases [1]. Research on dementia and AD has made significant progress in understanding disease mechanisms. However, still no curative treatment is available. The identification and understanding of modifiable risk factors for dementia is therefore much needed [2,3]. Although the strongest known risk factors for the onset of dementia are age and genetic predisposition, environmental factors such as diet quality seem to play a role even in older adults [[4], [5], [6]]. Therefore, even in a future where disease-modifying treatments are widespread, preventive measures will likely remain important.

Regarding diet quality, epidemiological studies are shifting from single nutrient assessments to dietary patterns. This approach can account for complex interrelationships between different foods and nutrients and therefore provide better insights into how diet might be associated with cognitive decline and brain pathology [7]. Several studies have shown that adherence to diets with low inflammatory potential, such as the Mediterranean diet (characterized by high intake of monounsaturated fat, fish, fruit, vegetables, cereals, and legumes, and low consumption of meat and dairy), is associated with larger brain volumes, increased glucose metabolism in the central nervous system, better cognitive function, and decreased accumulation of Aβ in the brain [8]. In contrast, unhealthy dietary patterns (such as the Western diet or inflammatory dietary indices) and inflammatory nutrient patterns (e.g., a diet high in saturated fatty acids, trans fats, or glycemic index) have been associated with smaller brain volumes [9], higher Aβ deposition [5,10] and higher risk of dementia [[11], [12], [13]].

While growing evidence suggests that adherence to different dietary patterns may modulate inflammation pathways, vascular disfunction, and oxidative stress in the central nervous system as in the rest of the body [5], comprehensive data from human studies is lacking and the underlying biological mechanisms are still not fully understood [4,10,14,15]. Although many studies have assessed the association of adherence to dietary patterns with neuroimaging and cerebrospinal fluid (CSF) AD biomarkers, only a few have focused on blood-based biomarkers [15]. The latter can be either specific to AD (e.g., phosphorylated tau 181 [p-tau181] and 42-aminoacid β amyloid peptide [Aβ-42]) or non-specific (e.g., total tau [t-tau], neurofilament light [NfL], and glial fibrillary acidic protein [GFAP]), have been shown to correlate with CSF and cerebral positron emission tomography (PET) biomarkers of AD pathology, and may be used to predict both all-cause dementia and AD-specific dementia [16,17]. A better understanding of the link between diet and the pathophysiological bases of dementia could pave the way for preventive dietary interventions against brain aging that can be monitored and evaluated in routine clinical care.

Accordingly, we performed a cross-sectional, population-based study of the association between two of the dietary patterns most consistently linked to inflammation and brain health (Mediterranean diet and inflammatory potential of diet) [9,11] and selected serum biomarkers of AD (t-tau, p-tau181, Aβ-42/40 ratio, NfL, and GFAP) among cognitively healthy older adults. We estimated adherence to the Mediterranean diet score [MDS] and Empirical dietary inflammatory index [EDII] in the main analyses and to alternative scores in sensitivity analyses. We hypothesized that the former and latter dietary patterns would be associated with lower and higher levels of blood-based biomarkers of AD, respectively.

## 2 Methods and materials

### 2.1 Study design and population

We used data from the population-based Swedish National study on Aging and Care in Kungsholmen (SNAC-K). It is an ongoing cohort study which recruited 3363 participants aged 60 years or older in 2001–2004 (participation rate 73%). The aim of the study was to better understand the process of aging and to identify possible preventive strategies to improve health and care in older adults. After the baseline examination, participants have been followed up regularly every 3 or 6 years, as described elsewhere [18]. This study includes baseline data only, as biomarkers of AD were not measured over the follow-up.

Institutionalized participants (n = 191) and those with dementia (n = 322) or Parkinson's disease (n = 40) were excluded. We also excluded participants with missing data on diet (n=580), serum biomarkers of AD (n=707), and sociodemographic variables (n=22) − note that one participant could lack data in more than one variable. After managing exclusions, our study sample consisted of 1907 older adults (Supplementary Fig. 1).

The protocol of the SNAC-K study was approved by the Regional Ethical Review Board in Stockholm. All participants provided written informed consent to participate. The results of this study are reported following the STROBE recommendations [19].

### 2.2 Study variables and data collection

#### 2.2.1 Dietary patterns

We used data from a validated 98-item food frequency questionnaire (FFQ) [20]. Participants indicated how frequently they consumed foods and drinks in the previous year by using a nine-point scale, ranging from ‘never or less than once a year’ to ‘more than 4 times a day’. Color photographs were used to estimate portion sizes, and food composition tables from the Swedish National Food Agency to estimate nutrient intakes. We used data on food groups, individual foods, and individual nutrients to estimate adherence to two dietary patterns: the Mediterranean Diet Score [21] and the Empirical Dietary Inflammatory Index [22]. The selection of two widely used dietary patterns may facilitate an easier comparison of results across existing studies.

##### 2.2.1.1 Mediterranean diet score

A scale indicating adherence to the traditional Mediterranean diet was built following the approach of Trichopoulou et al. [21]. We combined individual foods/nutrients from the FFQ into nine groups and calculated the average consumption (grams per day) for each group. A value of 0 or 1 was assigned to each of the nine groups, with the sex-specific median as the cutoff. For the beneficial components (vegetables, legumes, fruits and nuts, cereals, fish, and ratio of monounsaturated to saturated fatty acids), persons whose consumption was at or above the median were assigned a value of 1. Conversely, for the components presumed to be detrimental (meat/poultry and dairy products), persons whose consumption was below the median were assigned a value of 1. For alcohol intake, a value of 1 was assigned to men who had between 10 and 50 g of ethanol per day and to women who had between 5 and 25 g per day. Thus, the MDS ranged from 0 (minimal adherence to the traditional Mediterranean diet) to 9 (maximal adherence). Information on the consumption of the components and scoring of the MDS is shown in Supplementary Table 1.

##### 2.2.1.2 Empirical dietary inflammatory index

To estimate adherence to the EDII, we used a scoring method proposed by Tabung et al. [22], which identified the dietary pattern most predictive of three plasma inflammatory markers: interleukin-6 (IL-6), C-reactive protein (CRP), and tumor necrosis factor α receptor 2 (TNFαR2). In our study, we combined individual foods from the FFQ into 17 food groups and calculated the mean consumption (servings per day) for each group. Processed meat, red meat, organ meat, fish (other than dark-meat fish), vegetables (other than dark yellow vegetables, leafy green vegetables, and tomatoes), refined grains, high-energy beverages, and tomatoes were considered pro-inflammatory, while dark yellow vegetables, leafy green vegetables, snacks, fruit juice, pizza, tea, coffee, beer, and wine were deemed anti-inflammatory. The mean daily consumption of each food group by each participant was multiplied by an inflammatory effect score, ranging from -1175 (maximally anti-inflammatory) to 252 (maximally pro-inflammatory). Finally, the weighted consumption was summed to obtain the EDII, which was divided by 1000 to aid in interpretation. A higher EDII indicates a more pro-inflammatory diet and vice versa. Inflammatory effect scores of the components of the EDII and their consumption are shown in Supplementary Table 2.

#### 2.2.2 Blood-based biomarkers of AD

In SNAC-K, peripheral venous blood samples were collected (note that fasting was not mandatory). After centrifugation, serum aliquots were stored at the Karolinska Institutet Biobank at -80 °C in cryogenic storage vials for subsequent analysis. AD blood biomarker quantification was performed at the Affinity Proteomics Stockholm Unit (SciLifeLab).

Biomarkers were measured by Single Molecule Array technology (Simoa, Quanterix). Serum NfL and GFAP were assessed using the Simoa Neuro 2-plex B Kit; serum Aβ-40, Aβ-42, and t-tau were measured using the Simoa Neuro 3-plex A Kit; and for serum p-tau181 quantification, the Simoa pTau-181 Advantage V2 Kit was used. For each kit, 25 μL of the sample was diluted 1:4, and the assays were conducted following the manufacturer's instructions. The Quanterix instrument provided average enzyme per bead values for calibrators, controls, and samples. Curve-fitting, extrapolation of concentrations, and graphical representation were automatically performed with the Quanterix SR-X software, using the calibrators and a four-parameter logistic curve fit. Data below the limit of detection were replaced using a not-missing-at-random strategy, with an imputed value of 0 (imputed measurements in the whole cohort: n=6 for Aβ-42, 15 for p-tau181, and 15 for t-tau).

The Aβ42/40 ratio and p-tau181 are AD-specific biomarkers; a low Aβ42/40 ratio and elevated levels of p-tau181 point to the presence of AD-related neuropathological changes (i.e., amyloid plaques and neurofibrillary tangles). Elevated levels of t-tau are indicative of neuronal damage and commonly associated with AD as well as other neurodegenerative conditions. NfL is a nonspecific marker of neurodegeneration. It is a protein found in neurons and released into the cerebrospinal fluid and blood when neurons are damaged or die. The levels of NfL are elevated in various types of dementia. Lastly, GFAP is protein found in brain glial cells – astrocytes. Elevated levels are a marker of neuroinflammation [23].

#### 2.2.3 Other variables

We used data on several potential confounders of the association between dietary patterns and serum biomarkers of AD (Supplementary Fig. 2). We grouped potential confounders into (1) sociodemographic variables: sex, age, longest held occupation (manual worker or not), and highest educational level (elementary school, high school, or university); (2) lifestyle variables: smoking status (have never smoked, former smoker, current smoker, or no data), physical activity level (four categories, according to moderate and vigorous physical activity, or no data), body mass index (BMI) (<20, 20 to <25, 25 to <30, ≥30 kg/m^2^, or no data), and energy intake (kcal/day); (3) morbidity: diabetes, heart diseases (atrial fibrillation, heart failure, ischemic heart disease, or heart valve disease), cerebrovascular disease, chronic lung disease (chronic obstructive pulmonary disease, emphysema, or chronic bronchitis), cancer (hematological and solid neoplasms), depression and mood diseases, hypertension, anemia, and chronic kidney disease.

Data on sociodemographic variables, smoking status, and physical activity were obtained through face-to-face interviews performed by nurses and self-reported questionnaires. Weight and height were measured using standard protocols. Morbidity was diagnosed in accordance with standard procedures, based on participants’ medical history, examinations performed by physicians, participants’ and/or proxies’ interviews, diagnostic tests including instrumental and laboratory tests, and use of medications. Inpatient and outpatient medical records were also considered. The full methodology used for the classification of chronic diseases has been described in detail elsewhere [24].

In addition, DNA was extracted from peripheral blood samples and apolipoprotein E (*APOE*) was genotyped in 1862 participants. They were considered *APOE-ε4* carriers if they had at least one ε4 allele. Global cognition was assessed with the Mini-Mental State Examination (MMSE) [25].

### 2.3 Statistical analyses

#### 2.3.1 Descriptive analyses

Characteristics of the study sample were tabulated across tertiles of adherence to the MDS and EDII. Differences in socio-demographic, lifestyle, morbidity, and other characteristics were examined by using analysis of variance for continuous variables and χ^2^ tests for categorical variables.

#### 2.3.2 Main analyses

Due to the skewed distribution of the measured biomarkers and the substantial differences in their concentrations, we used bootstrapped quantile regression models to evaluate the association between adherence to the dietary patterns and standardized concentrations of blood-based biomarkers of AD. These models estimated the 25th, 50th and 75th quantiles of the dependent variable distribution (i.e., a given biomarker), conditional on the values of the independent variables (i.e., a given dietary pattern and potential confounders). Evaluating the study associations at different percentiles of the biomarkers’ distributions allowed to relax the assumption that the effects of diet on AD are the same at all levels of AD pathology. Confidence intervals were obtained via bootstrapping, and the variance-covariance matrix estimation included between-quantile blocks. To control for potential confounding, three incrementally adjusted models were used: (1) adjusted for sociodemographic characteristics, (2) additionally adjusted for lifestyle variables, and (3) further adjusted for morbidity. The adherence to the MDS and EDII was modelled as a continuous variable [per 1-standard deviation (SD) increment]. The associations between dietary patterns and plasma biomarkers were summarized with beta coefficients and their two-sided 95% confidence intervals (CI). Differences in the associations of a given dietary pattern with an AD biomarker across the 25th, 50th, and 75th percentiles of the biomarker distribution were computed as linear combinations of coefficients with the *lincom* command in Stata® (StataCorp LLC), version 17.0. The significance level was set at 0.05 for all statistical tests, which were two-tailed.

#### 2.3.3 Subgroup analyses and interactions

We conducted several subgroup analyses using fully adjusted quantile regression models estimating the 50th percentile of the biomarkers’ distributions. Confidence intervals were also obtained via bootstrapping. We stratified the associations by three well-known nonmodifiable risk factors of AD [4] – sex (males and females), age (<78 and ≥78 years, according to the SNAC-K study design [18]), and *APOE-ε4* genotype (non-carriers and carriers). Estimates for each stratum were obtained from models with multiplicative interaction terms between the dietary patterns and the stratification variables. This approach aimed to minimize power loss in subgroup analyses under the assumption that the effects of dietary patterns could change over sex, age, or *APOE-ε4* genotype while remaining constant over the levels of potential confounders.

#### 2.3.4 Sensitivity analyses

On the one hand, we studied the associations of the individual components of the MDS and EDII with the AD biomarkers. These models closely resembled those used in the main analyses, but were adjusted for all other components of the score being examined. On the other hand, we assessed the robustness of study associations severalfold. Because cognitive impairment might influence the reporting of food consumption, we performed a sensitivity analysis in which we excluded participants with MMSE below 27. Given that the MDS could estimate relative instead of absolute adherence to the Mediterranean diet, we recalculated the score based on the sex-specific consumption of the MDS components in a Greek population [21]. Since some of the EDII foundations may diverge from other healthy dietary patterns (including the MDS), we calculated alternative versions of the EDII with pro-inflammatory scoring for snacks, beer, and pizza; and with anti-inflammatory scoring for fish (other than dark-meat fish), other vegetables (i.e., vegetables other than leafy green vegetables and dark yellow vegetables), and tomatoes. We also assessed diet quality with the Alternative Mediterranean Diet (AMED) [26] and Dietary Inflammatory Index (DII) [27], as opposed to the MDS and EDII. Finally, we evaluated whether a healthy, conceptually different dietary pattern (i.e., Dietary Approaches to Stop Hypertension [DASH] [28]) was associated with AD biomarkers.

## 3 Results

### 3.1 Description of study participants

**Fig. 1:**
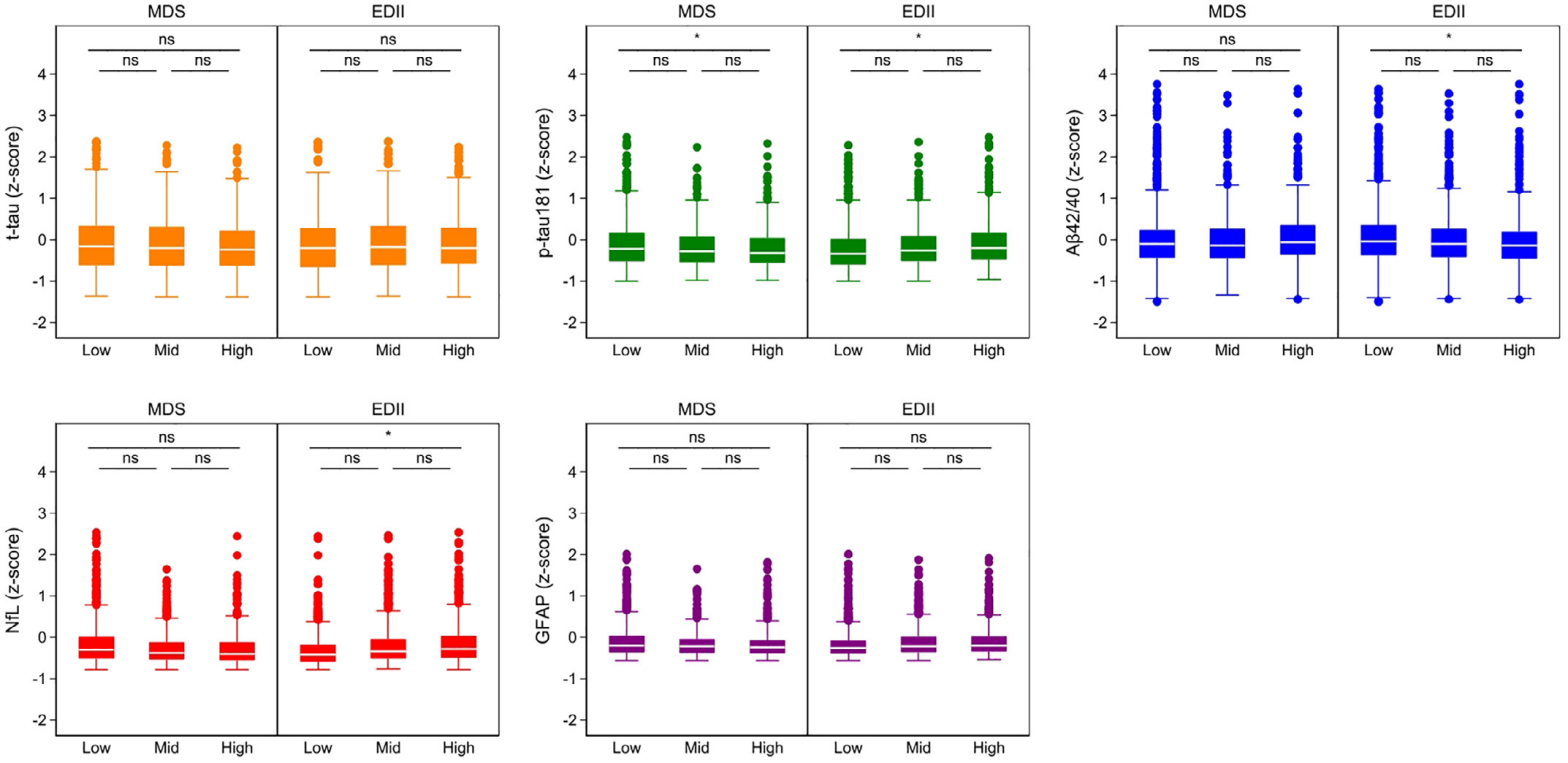
Distribution of the blood-based biomarkers’ levels by tertiles of adherence to the Mediterranean diet score (MDS) and Empirical Dietary Inflammatory Index (EDII) (n = 1907). * p < 0.05; ns: non‐significant; Aβ40 = 40-aminoacid β amyloid peptide; Aβ42 = 42-aminoacid β amyloid peptide; t-Tau = total tau; p-Tau181 = phosphorylated tau 181; NfL = neurofilament light; GFAP = glial fibrillary acidic protein Adherence to MDS categories: (i) lower: ≤ 3, (ii) mid: 4 or 5, (iii) higher: ≥ 6; adherence to EDII categories: (i) lower: ≤-0,079, (ii) mid: -0.078 to 0.113, (iii) higher: ≥ 0.113 Z-scores were plotted for adherences to the dietary patterns above the 1st percentile and below the 99th percentile.

The characteristics of study participants are reported in Table 1. Those with lower adherence to MDS or higher adherence to EDII (i.e., unhealthier diet) were older, more likely lifelong manual workers and living alone, had a lower educational level, more likely to have chronic kidney disease, and worse cognitive function. The distribution of serum biomarkers of AD across tertiles of the MDS and EDII is shown in Fig. 1.

**Table 1:** Characteristics of the study participants by tertiles of adherence to the Mediterranean Diet Score (MDS) and Empirical Dietary Inflammatory Index (EDII) (n = 1907).

	MDS				EDII			
	Lower (n=518)	Mid (n=883)	Higher (n=506)		Lower (n=637)	Mid (n=635)	Higher (n=635)	
***Socio-demographic variables***								
Sex, male [n(%)]	211 (40.7)	346 (39.1)	199 (39.3)		257 (40.4)	246 (38.7)	252 (39.7)	
Age [Median (IQR)]	72.15 (60.9, 78.7)	67.0 (60.7, 78.3)	66.3 (60.5, 72.6)	*	66.2 (60.5, 72.5)	72.1 (60.9, 78.2)	72.2 (66.1, 81.1)	*
Manual worker [n (%)]	118 (22.8)	165 (18.7)	80 (15.8)	*	89 (14.0)	128 (20.2)	146 (23.0)	*
Education level [n (%)]								
Elementary	81 (15.6)	115 (13.0)	44 (8.7)	*	59 (9.3)	78 (12.3)	103 (16.2)	*
High school	262 (50.6)	445 (50.4)	228 (45.1)		270 (42.4)	332 (52.3)	333 (52.4)	
University	175 (33.8)	323 (36.6)	234 (46.3)		308 (48.4)	225 (35.4)	199 (31.3)	
***Lifestyle variables***								
Tobacco smoking [n (%)]				*				
Never	212 (40.9)	389 (44.1)	220 (43.5)		233 (36.6)	295 (46.5)	293 (46.1)	*
Former	215 (41.5)	342 (38.7)	221 (43.7)		275 (43.2)	241 (38.0)	262 (41.3)	
Current	90 (17.4)	149 (16.9)	59 (11.7)		126 (19.8)	96 (15.1)	76 (12.0)	
Physical activity level [n (%)]								
Sedentary	119 (23.0)	172 (20.7)	66 (13.9)	*	105 (16.5)	136 (21.4)	116 (18.3)	
Low active	182 (35.1)	331 (39.7)	193 (40.6)		259 (40.7)	230 (36.2)	217 (34.2)	
Active	146 (28.2)	275 (33.0)	180 (37.8)		192 (30.1)	202 (31.8)	207 (32.6)	
Very active	25 (4.8)	55 (6.6)	37 (7.8)		43 (6.8)	31 (4.9)	37 (5.8)	
BMI (kg/m^2^) [n (%)]								
< 20	31 (6.0)	30 (3.4)	22 (4.4)		29 (4.6)	25 (3.9)	29 (4.6)	
20 to <25	207 (40.0)	362 (41.0)	193 (38.1)		257 (40.4)	259 (40.8)	246 (38.7)	
25 to <30	189 (36.5)	375 (42.5)	219 (43.3)		271 (42.5)	269 (42.4)	243 (38.3)	
≥ 30	86 (16.6)	112 (12.7)	66 (13.0)		75 (11.8)	79 (12.4)	110 (17.3)	
Energy intake (kcal/day) [Median (IQR)]	1617 (1282, 1942)	1786 (1445, 2176)	2077 (1703, 2457)	*	1716 (1379, 2086)	1757 (1434, 2121)	1996 (1606, 2455)	*
***Morbidities***								
Diabetes mellitus [n (%)]	47 (9.1)	64 (7.3)	45 (8.9)		28 (4.4)	51 (8.0)	77 (12.1)	*
Heart diseases [n (%)]	109 (21.0)	166 (18.8)	96 (19.0)		87 (13.7)	123 (19.4)	161 (25.4)	*
Cerebrovascular disease [n (%)]	26 (5.0)	41 (4.6)	16 (3.2)		24 (3.8)	28 (4.4)	31 (4.9)	
Cancer [n (%)]	38 (7.3)	59 (6.7)	43 (8.5)		38 (6.0)	46 (7.2)	56 (8.8)	
Depression [n (%)]	38 (7.3)	64 (7.3)	40 (7.9)		48 (7.5)	45 (8.5)	40 (6.0)	
Hypertension [n (%)]	357 (68.9)	602 (68.2)	351 (69.4)		408 (64.1)	445 (70.1)	457 (72.0)	*
Anemia [n (%)]	54 (10.4)	57 (6.5)	36 (7.1)	*	41 (6.4)	52 (8.2)	54 (8.5)	
COPD [n (%)]	31 (6.0)	33 (3.7)	14 (2.8)	*	21 (3.3)	20 (3.1)	37 (5.8)	
Chronic kidney disease [n (%)]	220 (42.5)	351 (39.8)	152 (30.0)	*	184 (28.9)	252 (39.5)	288 (45.4)	*
***Other variables***								
MMSE <27 [n (%)]	57 (11.2)	70 (8.1)	31 (6.2)	*	40 (6.4)	43 (6.8)	75 (12.1)	*
*APOE-ε4* [n (%)]	414 (28.0)	266 (30.8)	140 (28.4)		192 (30.6)	182 (29.5)	173 (28.0)	
***Biomarkers of Alzheimer's disease***								
t-tau (pg/ml)	0.8 (0.5, 1.2)	0.8 (0.5, 1.2)	0.8 (0.5, 1.1)		0.81 (0.5, 1.1)	0.8 (0.5, 1.2)	0.8 (0.6, 1.1)	
p-tau181 (pg/ml)	1.2 (0.7, 1.9)	1.1 (0.7, 1.7)	1.0 (0.7, 1.6)	*	1.0 (0.6, 1.5)	1.1 (0.7, 1.6)	1.2 (0.8, 1.8)	*
Aβ 40 / Aβ 42	0.057 (0.049, 0.066)	0.058 (0.049, 0.067)	0.060 (0.051, 0.069)		0.059 (0.050, 0.069)	0.058 (0.050, 0.067)	0.057 (0.048, 0.065)	*
NfL (pg/ml)	18.1 (12.6, 27.1)	16.9 (12.3, 23.9)	15.3 (11.5, 22.7)		14.9 (10.8, 21.4)	16.9 (12.5, 24.8)	18.5 (13.0, 26.8)	*
GFAP (pg/ml)	117.9 (79.0, 188.4)	114.5 (75.2, 165.7)	110.0 (72.7, 152.8)		103.7 (71.0, 152.5)	116.5 (79.5, 175.2)	120.5 (84.0, 179.9)	

*P value < 0.05 (two-sided) for differences in means (ANOVA) or proportions (Pearson's chi-squared) across categories of adherence to the MDS or EDII.

MDS: (i) lower: ≤ 3; (ii) mid: 4 or 5; (iii) higher: ≥ 6; EDII: (i) lower: ≤-0,079; (ii) mid: -0.078 to 0.113; (iii) higher: ≥ 0.113; Physical activity: (i) Sedentary: less than 0.5 hours/day of moderate physical activity, (ii) Low active: ≥ 0.5 hours/day of moderate physical activity, (iii) Active: ≥ 1 hour/day of moderate physical activity, and (iv) Very active: ≥ 3 hours/day of moderate physical activity or ≥ 1 hour/day of moderate and ≥ 1 hour/day of vigorous physical activity.

BMI: body mass index; IQR: interquartile range; COPD: chronic obstructive pulmonary disorder; MMSE: Mini Mental State Examination. Aβ40 = 40-aminoacid β amyloid peptide; Aβ42 = 42-aminoacid β amyloid peptide; t-Tau = total tau; p-Tau181 = phosphorylated tau 181; NfL = neurofilament light; GFAP = glial fibrillary acidic protein.

Missing data: tobacco smoking (n=10), physical activity level (n=127), BMI (n=15), APOE-E4 (n=45).

### 3.2 Main results

In the fully-adjusted models, higher adherence to the MDS was associated with lower levels of p-tau181 at the 50th and 75th percentiles [model 3 β (95% CI) per 1-SD increment = -0.028 (-0.053, -0.002) and -0.036 (-0.072, -0.001), respectively] (Table 2, Supplementary Figs. 4 and 5). Associations between the MDS and p-tau181 were significantly weaker at the 25th percentile of the biomarker's distribution (Supplementary Table 3). Adherence to the EDII was not associated with the levels of p-tau181.

**Table 2:** Associations between the Mediterranean Diet Score (MDS) and Empirical Dietary Inflammatory Index (EDII) (per 1-SD increment) and levels of blood-based biomarkers of Alzheimer's disease (at the 25th, 50th, and 75th percentiles).

	MDS β (95% CI)			EDII β (95% CI)		
Biomarker	25th percentile	50th percentile	75th percentile	25th percentile	50th percentile	75th percentile
**t-tau**						
Model 1	-0.001 [-0.034, 0.032]	-0.007 [-0.049, 0.035]	-0.024 [-0.074, 0.026]	0.002 [-0.030, 0.033]	-0.026 [-0.065, 0.014]	-0.010 [-0.059, 0.039]
Model 2	-0.002 [-0.036, 0.032]	-0.006 [-0.058, 0.046]	-0.013 [-0.066, 0.039]	0.003 [-0.033, 0.040]	-0.018 [-0.063, 0.028]	0.000 [-0.052, 0.053]
Model 3	-0.007 [-0.042, 0.028]	0.016 [-0.033, 0.065]	-0.016 [-0.075, 0.043]	-0.015 [-0.047, 0.017]	-0.019 [-0.064, 0.026]	0.009 [-0.044, 0.061]
**p-tau181**						
Model 1	0.003 [-0.021, 0.027]	-0.023 [-0.045, -0.001]*	-0.023 [-0.045, -0.000]*	0.017 [-0.002, 0.036]	0.014 [-0.010, 0.038]	0.039 [0.003, 0.076]*
Model 2	-0.002 [-0.027, 0.024]	-0.031 [-0.055, -0.006]*	-0.051 [-0.087, -0.014]**	0.016 [0.000, 0.031]*	0.018 [-0.010, 0.045]	0.038 [-0.006, 0.083]
Model 3	-0.003 [-0.029, 0.022]	-0.028 [-0.053, -0.002]*	-0.036 [-0.072, -0.001]*	0.015 [-0.004, 0.034]	0.003 [-0.023, 0.029]	0.017 [-0.026, 0.059]
**Aβ 42/40**						
Model 1	0.020 [-0.008, 0.048]	0.008 [-0.017, 0.034]	0.008 [-0.018, 0.035]	-0.007 [-0.033, 0.018]	-0.017 [-0.052, 0.017]	-0.022 [-0.062, 0.018]
Model 2	0.030 [-0.001, 0.060]	0.009 [-0.022, 0.039]	0.016 [-0.029, 0.060]	-0.007 [-0.037, 0.023]	-0.011 [-0.035, 0.013]	-0.019 [-0.068, 0.029]
Model 3	0.035 [0.003, 0.067]*	0.002 [-0.028, 0.032]	0.013 [-0.030, 0.056]	-0.003 [-0.033, 0.027]	-0.013 [-0.036, 0.011]	-0.018 [-0.066, 0.030]
**NfL**						
Model 1	-0.012 [-0.023, -0.000]*	-0.010 [-0.023, 0.002]	-0.010 [-0.022, 0.001]	0.006 [-0.007, 0.019]	0.006 [-0.010, 0.023]	0.020 [-0.002, 0.041]
Model 2	-0.010 [-0.021, 0.002]	-0.011 [-0.026, 0.003]	-0.008 [-0.027, 0.011]	0.011 [-0.002, 0.024]	0.013 [-0.002, 0.027]	0.025 [0.002, 0.049]*
Model 3	-0.015 [-0.027, -0.002]*	-0.009 [-0.024, 0.005]	-0.003 [-0.025, 0.019]	0.007 [-0.005, 0.020]	0.012 [-0.002, 0.027]	0.031 [0.008, 0.053]*
**GFAP**						
Model 1	-0.002 [-0.011, 0.008]	-0.002 [-0.015, 0.012]	-0.002 [-0.013, 0.009]	0.000 [-0.013, 0.013]	0.006 [-0.007, 0.018]	0.004 [-0.016, 0.024]
Model 2	-0.003 [-0.014, 0.008]	-0.006 [-0.018, 0.006]	-0.008 [-0.031, 0.014]	0.001 [-0.011, 0.013]	0.002 [-0.011, 0.014]	0.005 [-0.016, 0.026]
Model 3	-0.001 [-0.012, 0.010]	-0.003 [-0.015, 0.009]	-0.003 [-0.024, 0.019]	0.000 [-0.011, 0.011]	0.005 [-0.007, 0.017]	0.000 [-0.022, 0.022]

*p < 0.05; **p < 0.01; CI (confidence interval); Aβ40 = 40-aminoacid β amyloid peptide; Aβ42 = 42-aminoacid β amyloid peptide; t-Tau = total tau; p-Tau181 = phosphorylated tau 181; NfL = neurofilament light; GFAP = glial fibrillary acidic protein

Range of the dietary patterns: MDS: 0 to 9, 1-SD increment, 1.60; EDII: -1.106 to 2.773, 1-SD increment, 0.30

Range of the biomarkers (standardized): t-tau: -1.42 to 23.65; p-tau181: -1.03 to 15.45; Aβ 40/Aβ 42: -2.36 to 23.04; NfL: 2.36 to 23.04; GFAP: -0.62 to 23.49

Biomarkers’ percentile values (standardized): t-tau (25th: -0.628, 50th: -0.193, 75th: 0.315); p-181 (25th: -0.544, 50th: -0.274, 75th: 0.116); Aβ 40/Aβ 42 (25th: -0.427, 50th: -0.095, 75th: 0.271); NfL (25th: -0.534, 50th: -0.353, 75th: -0.039), GFAP (25th: -0.375, 50th: -0.224, 75th: -0.012).

Model 1: bootstrapped quantile regression model adjusted for sex, age, living arrangements (living alone or not), longest held occupation (manual worker or not), and highest educational level (elementary school, high school, or university).

Model 2: as Model 1, and additionally adjusted for smoking status (have never smoked, former smoker, current smoker, or no data), physical activity level (sedentary, low active, active, very active, no data), body mass index (<20, 20 to <25, 25 to <30, ≥30 kg/m^2^, or no data), and energy intake (kcal/day).

Model 3: as Model 2, and further adjusted for diabetes, heart diseases (atrial fibrillation, heart failure, ischemic heart disease, or heart valve disease), cerebrovascular disease, chronic lung disease (chronic obstructive pulmonary disease, emphysema, or chronic bronchitis), cancer (hematological and solid neoplasms), depression and mood diseases, hypertension, anemia, and chronic kidney disease.

A significant association between adherence to the MDS and a higher (positive) Aβ42/40 ratio was apparent at lower levels of the biomarker's distribution (i.e., 25th percentile) [0.035 (0.003, 0.067)] (Table 2, Supplementary Fig. 3). This association was significantly weaker at higher levels of the biomarker (Supplementary Table 3). No associations between the EDII and the Aβ42/40 ratio were observed.

Both dietary patterns showed significant associations with NfL (Table 2, Supplementary Figs. 3 and 5). While higher adherence to MDS was associated with lower levels of NfL at lower levels of its distribution (i.e., 25th percentile) [-0.015 (-0.027, -0.002)], an association between higher EDII and higher levels of NfL was detected at higher levels of the biomarker's distribution (i.e., 75th percentile) [0.031 (0.008, 0.053)]. The association between the EDII and NfL was significantly weaker at lower levels of the biomarker's distribution (Supplementary Table 3).

Neither the MDS nor the EDII were significantly associated with t-tau or GFAP at the 25th, 50th, or 75th percentiles of their distribution (Table 2, Supplementary Figs. 3–5).

### 3.3 Subgroup and sensitivity analyses

Results of subgroup analyses are shown in Table 3. The inverse association between the MDS and p-tau181 was not significantly different in males and females or in the youngest and oldest old, but was stronger in *APOE-ε4* carriers compared to non-carriers [-0.065 (-0.106, -0.024) and -0.018 (-0.050, 0.015); p-interaction = 0.008]. An inverse association between the MDS and GFAP arose in participants ≥78 years, but not in those aged <78 [-0.042 (-0.077, -0.007) and 0.006 (-0.008, 0.020); p-interaction = 0.014]. When analyzing adherence to the EDII, no significantly different associations across subgroups were observed.

**Table 3:** Associations between the Mediterranean Diet Score (MDS) and Empirical Dietary Inflammatory Index (EDII) (per 1-SD increment) and levels of blood-based biomarkers of Alzheimer´s disease (at the 50th percentile), stratified by sex, age, and APOE-e4 genotype.

SEX						
	MDS β (95% CI)			EDII β (95% CI)		
Biomarker	Male (n=755)	Female (n=1152)	p-interaction	Male (n=755)	Female (n=1152)	p-interaction
t-tau	0.010 [-0.073, 0.094]	0.026 [-0.031, 0.082]	0.776	-0.043 [-0.107, 0.021]	-0.006 [-0.059, 0.047]	0.361
p-tau181	-0.036 [-0.081, 0.009]	-0.025 [-0.054, 0.003]	0.699	0.013 [-0.029, 0.056]	0.001 [-0.029, 0.030]	0.617
Aβ 42/40	-0.004 [-0.051, 0.044]	0.004 [-0.031, 0.037]	0.823	-0.027 [-0.081, 0.028]	0.006 [-0.035, 0.048]	0.336
NfL	-0.015 [-0.041, 0.011]	-0.006 [-0.023, 0.011]	0.542	0.004 [-0.016, 0.025]	0.016 [-0.001, 0.033]	0.377
GFAP	-0.006 [-0.025, 0.013]	-0.006 [-0.023, 0.012]	0.984	0.004 [-0.016, 0.025]	0.006 [-0.008, 0.020]	0.910

**AGE**						

	**MDS β (95% CI)**			**EDII β (95% CI)**		
**Biomarker**	**<78 years (n=1306)**	**≥ 78 years (n=601)**	**p-interaction**	**<78 years (n=1306)**	**≥ 78 years (n=601)**	**p-interaction**

t-tau	-0.019 [-0.076, 0.038]	0.048 [-0.039, 0.135]	0.193	-0.010 [-0.064, 0.044]	-0.019 [-0.114, 0.075]	0.874
p-tau181	-0.033 [-0.064, -0.003]*	-0.029 [-0.081, 0.022]	0.899	0.004 [-0.025, 0.033]	0.005 [-0.063, 0.074]	0.977
Aβ 42/40	0.007 [-0.036, 0.050]	-0.002 [-0.051, 0.048]	0.809	-0.015 [-0.054, 0.024]	0.015 [-0.046, 0.076]	0.456
NfL	-0.001 [-0.014, 0.011]	-0.044 [-0.088, -0.001]*	0.061	0.011 [-0.004, 0.026]	0.011 [-0.027, 0.049]	0.995
GFAP	0.006 [-0.008, 0.020]	-0.042 [-0.077, -0.007]**	**0.014**	0.007 [-0.004, 0.018]	-0.003 [-0.034, 0.028]	0.538

***APOE-ε4***						

	**MDS β (95% CI)**			**EDII β (95% CI)**		
**Biomarker**	**Non carrier (n= 1315)**	**Carrier (n=547)**	**p-interaction**	**Non carrier (n= 1315)**	**Carrier (n=547)**	**p-interaction**

t-tau	0.017 [-0.047, 0.081]	-0.007 [-0.082, 0.068]	0.627	-0.048 [-0.105, 0.010]	0.000 [-0.081, 0.081]	0.333
p-tau181	-0.018 [-0.050, 0.015]	-0.065 [-0.106, -0.024]***	**0.008**	-0.004 [-0.029, 0.020]	0.019 [-0.034, 0.072]	0.440
Aβ 42/40	-0.011 [-0.048, 0.027]	0.025 [-0.023, 0.073]	0.241	-0.005 [-0.042, 0.031]	-0.057 [-0.117, -0.003]	0.132
NfL	-0.020 [-0.038, -0.002]**	0.002 [-0.020, 0.024]	0.108	0.020 [0.005, 0.035]***	-0.009 [-0.039, 0.022]	0.080
GFAP	-0.005 [-0.021, 0.012]	-0.008 [-0.037, 0.021]	0.844	0.005 [-0.010, 0.020]	0.007 [-0.014, 0.029]	0.851

*p < 0.05; **p < 0.01; ***p < 0.001; CI (confidence interval); Aβ40 = 40-aminoacid β amyloid peptide; Aβ42 = 42-aminoacid β amyloid peptide; t-Tau = total tau; p-Tau181 = phosphorylated tau 181; NfL = neurofilament light; GFAP = glial fibrillary acidic protein

Range of the dietary patterns: MDS: 0 to 9, 1-SD increment, 1.60; EDII: -1.106 to 2.773, 1-SD increment, 0.30

Range of the biomarkers (standardized): t-tau: -1.42 to 23.65; p-tau181: -1.03 to 15.45; Aβ 40 / Aβ 42: -2.36 to 23.04; NfL: 2.36 to 23.04; GFAP: -0.62 to 23.49

Biomarkers’ values at 50th percentile (standardized): t-tau: -0.193; p-181: -0.274; Aβ 40 / Aβ 42: -0.095; NfL: -0.353; GFAP: -0.224

Quantile regression models adjusted as Model 3 in Table 2: sex, age, living arrangements (living alone or not), longest held occupation (manual worker or not), highest educational level (elementary school, high school, or university), smoking status (have never smoked, former smoker, current smoker, or no data), physical activity level (sedentary, low active, active, very active, no data), body mass index (<20, 20 to <25, 25 to <30, ≥30 kg/m^2^, or no data), energy intake (kcal/day), diabetes, heart diseases (atrial fibrillation, heart failure, ischemic heart disease, or heart valve disease), cerebrovascular disease, chronic lung disease (chronic obstructive pulmonary disease, emphysema, or chronic bronchitis), cancer (hematological and solid neoplasms), depression and mood diseases, hypertension, anemia, and chronic kidney disease.

Estimates were obtained from models with multiplicative interaction terms between the dietary patterns and the stratification variables.

The direction of the study associations did not generally change (although their strength was sometimes reduced) when: 1) excluding participants with MMSE below 27 (n=158); 2) recalculating the MDS to reflect absolute as opposed to relative adherence to the Mediterranean diet; or 3) when computing alternative versions of the EDII with pro-inflammatory scoring for snacks, beer, and pizza; and 4) anti-inflammatory scoring for other fish, tomatoes, and other vegetables (Supplementary Table 4). The individual components of the MDS and EDII were not generally associated with the AD biomarkers (Supplementary Tables 5 and 6).

When assessing diet quality in alternative ways, higher adherence to the AMED was associated with lower levels of t-tau and p-tau181 and higher (positive) levels of the Aβ 42/40 ratio. Higher adherence to the DII was associated with lower (negative) levels of the Aβ 42/40 ratio. All associations were observed at the 25th percentile of the biomarkers’ distribution (Supplementary Table 7). Regarding the DASH, it was associated with higher levels of t-tau at the 25th percentile of the biomarker's distribution and with lower levels of p-tau 181 at the 25th and 75th percentiles of its distribution (Supplementary Table 8).

## 4 Discussion

In this cross-sectional study, we showed a consistent association between higher adherence to the MDS and lower levels of p-tau181 and an association between higher adherence to the EDII and higher levels of NfL at medium and/or high levels of said biomarkers. Higher adherence to the MDS was also associated to a lower Aβ42/40 ratio and elevated NfL levels, but only at lower levels of the biomarkers. Certain associations between diet quality and AD biomarkers were only apparent among the oldest old (i.e., MDS and lower levels of GFAP) and stronger in *APOE-ε4* carriers (i.e., MDS and lower levels of p-tau181).

### 4.1 Interpretation

Data on the association between dietary patterns and blood-based biomarkers of AD are limited, as most studies have analyzed CSF or neuroimaging biomarkers [6,29,30]. To our knowledge, there is only one published study on dietary patterns and blood-based biomarkers of AD, which is focused on their relationship with cognitive impairment. This study was conducted among postmenopausal women [15]. The most relevant findings of investigations on dietary patterns and AD and AD biomarkers are discussed below.

Our finding of lower levels of p-tau181 and higher Aβ42/40 ratio among the participants with higher adherence to the MDS are aligned with previous research investigating other biological fluids or brain imaging, which shows associations between Mediterranean dietary patterns and lower AD burden (i.e., amyloid-β and tau tangles) [29]. It is of note that associations of the Mediterranean diet with p-tau181 had not been reported before in observational or intervention studies [31,32]. First, in a recent 4-week randomized controlled trial by Hoscheidt et al., including participants aged 45 to 65 years with normal cognition (n=56) and mild cognitive impairment (n=31), neither a Western-like nor a Mediterranean-like diet affected CSF p-tau181 levels in any subgroup of participants [31]. Worth considering are the short intervention period, younger population, and small sample size of the study, which could have made diet's impact on tau pathology less apparent. Second, in a European multicenter cross-sectional study (n= 1625), no association between the Mediterranean diet and CSF p-tau181 was recorded in either Mediterranean or non-Mediterranean regions [32]. Possible explanations for the differences with our results include an alternative operationalization of the Mediterranean diet, a diverse geographical distribution (which could have increased the variability in dietary habits and diluted the study associations), and the inclusion of participants with preclinical AD and mild cognitive impairment. In our study, we only included cognitively intact participants and found this association only when the biomarker's levels were higher.

A positive association between adherence to the MDS and Aβ42/40 ratio at lower levels of the ratio distribution could be expected coupled with the strong association that was found with p-tau181 at higher levels of its distribution. Specifically, the presence of phosphorylated tau (i.e. p-tau181) in CSF or blood may not be only a consequence of neuronal damage, but also of its release in response to Aβ40 accumulation (which would result in a lower Aβ42/40 ratio) [33]. This association between adherence to the MDS and Aβ accumulation is supported by a previous cohort study in which a strong association between higher adherence to MDS and lower Aβ accumulation (assessed with PET) was found [30]. It must be emphasized that the corresponding association with Aβ42/40 ratio in our study was only marginal, and only at the 25th percentile of its distribution − even though the association was not statistically different from that at mid (50th percentile) and higher (75th percentile) levels. This rather weak association could be due to concurrent phenomena, such as peripheral amyloid production, hampering the interpretation of blood-based Aβ assays [17].

It is also of note that an inverse association between adherence to the MDS and levels of NfL was found only at lower levels of the biomarker's distribution. NfL is a biomarker of neurodegeneration, which appears to be the final chronological stage of AD pathology [33], but its increase can already be detected at its prodromal stages [16]. However, the presence of high levels of NfL in all neurodegenerative diseases makes this marker less specific for AD. The observed association may be due to the fact that the relationship between diet and neurodegeneration is more apparent in its early stages, while in more advanced stages it might be mediated by further biological mechanisms. To the best of our knowledge, ours is the first study to find an association between the MDS and NfL, so further investigation of the underlying mechanisms is needed.

Dietary inflammation may also play a role in neurodegeneration, as pro-inflammatory diets have previously been associated with poor cognitive performance, cognitive impairment, accelerated cognitive decline, and lower brain volume and cognitive function [5,9,13,34]. Our finding of a direct association between the EDII and NfL is also in line with a previous cross-sectional study in SNAC-K, in which nutrient patterns characterized by low intake of vegetables and high in processed meat and offal were associated with lower total brain volume and higher white matter hyperintensities volume, which are also markers of neurodegeneration [35]. The only published study investigating the association between inflammatory dietary patterns and AD blood-based biomarkers was that of Duggan et al. [15]. It demonstrated associations between adherence to the EDII, several inflammatory proteins, plasma biomarkers of AD pathology (Aβ-42/40) and neurodegeneration (NfL), and risk for dementia [15]. Our finding of an association between the EDII and NfL was consistent with Duggan's, but that between the EDII and Aβ-42/40 was not. This difference may be explained by the fact that said study sample was limited to white females, who present with higher Aβ42/40 than males [36].

In the present study, the relationships between dietary patterns and some AD biomarkers were more pronounced at higher levels of said biomarkers (50th and 75th percentiles), while other associations were only apparent at lower biomarker levels (25th percentile). On the one hand, associations with AD pathology biomarkers (e.g., ptau-181 and Aβ42/40) might be less pronounced in early disease stages due to small underlying biochemical changes that cannot be detected by these markers [37,38]. On the other hand, these results suggest that diet quality may have a higher influence on AD pathology when the latter is more pronounced. To the best of our knowledge, information about the dose-response relationship of the association between diet and AD biomarkers, either cross-sectionally or in the short-/long-term, is lacking. Accordingly, our cross-sectional findings and interpretations are only suggestive and need to be further explored in future longitudinal studies.

A possible reason for the observed inverse association between adherence to the MDS and p-tau181 could be the presence of dietary bioactive compounds in this dietary pattern. Previous studies have shown that molecules such as resveratrol, rutin, and myricetin, which are found in vegetables, fruits, and nuts, may ameliorate tau pathology in cell and animal models [39,40]. The potential impact of the MDS and EDII on AD blood biomarkers and cognitive decline may also be mediated by inflammatory pathways [14,41]. Inflammation is believed to activate toll-like receptors and receptors for advanced glycation end products, impair the function of the blood-brain barrier, reduce cerebral blood flow, and accelerate neuronal damage, all of which can increase the risk of cognitive decline [[42], [43], [44]]. Although inflammation has been suggested as the most influential pathway linking diet and AD pathology, other mechanisms could be at play. These include oxidative stress, insulin resistance, lipid metabolism dysregulation, and the gut-brain axis [4].

Explanations of the observed differences in study associations across age subgroups and *APOE-ε4* genotypes must be conjectural and interpreted with caution. We found that higher adherence to the MDS was associated with lower levels of GFAP only in those aged 78+, in whom every year of age was associated with a 0.014 z-score increase in GFAP levels. First, aging is the main risk factor for dementia, including AD, through specific biological hallmarks (e.g., glia-mediated neuroinflammation, mitochondrial disfunction) that accelerate pathology [45]. Nevertheless, these biological indicators can be influenced by diet, highlighting the overall impact of eating patterns on brain health [46,47]. Given the correlation between chronological and biological aging, it is possible that healthy dietary patterns may play a more substantial neuroprotective role in later stages of aging, once the hallmarks of aging have long been acting [45]. However, the discussed result is only suggestive, as we only found a significant age interaction in the association between one dietary pattern and one biomarker of AD. Our research sample was also limited to generally healthier individuals, which may reduce the external validity of our results. Moreover, no other study seems to have explored the associations between diet and GFAP yet, so our finding needs to be further investigated.

In addition, we found that *APOE-ε4* carriers showed a stronger association between higher adherence to the MDS and lower p-tau181 levels. It is of note that *APOE-ε4* is involved in several important roles in the central nervous system (i.e., cholesterol transport, neuroplasticity, and inflammation), and represents the strongest genetic risk factor for AD [48,49]. Previous research has shown that *APOE-ε4* carriers have more compromised neuroanatomical reserves, poorer brain protection, higher pro-inflammatory cytokine production, and worse repair mechanisms than persons without the *APOE-ε4* allele, which could make them more vulnerable to environmental factors affecting the brain [49].

Regarding sensitivity analyses, we observed similar associations with the AD biomarkers for the two alternate EDII versions with 1) pro-inflammatory scoring for snacks, beer, and pizza, and 2) anti-inflammatory scoring for other fish, tomatoes, and other vegetables. Any explanation for these findings is speculative in nature, though it possibly reflects food preparation methods. On one hand, well-done or browned fried, grilled, or barbecued fish may be proinflammatory -due to the oxidation of long-chain polyunsaturated fatty acids and the generation of heterocyclic amines and benzopyrene- [22]. On the other hand, while the effects of net tomato consumption on concentrations of inflammatory markers are conflictive, tomato paste contains 2.5- to 4-fold higher bioavailable lycopene than fresh tomatoes, which could explain the inverse association of pizza with inflammatory biomarkers, given the anti-inflammatory properties of lycopene [22]. These sensitivity analyses suggest that a maximally anti-inflammatory dietary pattern might not necessarily be optimal from the overall health perspective, so that the EDII may benefit from some modifications if it is to be used for AD pathology prevention.

### 4.2 Strengths and limitations

Strengths of this study include its population-based design, with a comprehensive collection of potential confounders and a large sample of older adults. Another advantage is the estimation of two antagonistic dietary patterns at the same time, which allowed side-by-side comparisons. Not focusing on single nutrients is in line with the paradigm shift in nutritional epidemiology in the last decades, as dietary patterns allow to focus on the entire diet and consider complex interrelationships between different foods and/or nutrients. Indeed, individual components of the analyzed dietary patterns were not generally associated with AD biomarkers, contrary to the patterns themselves. This may better reflect participant dietary habits and provide deeper insights on how diet might be associated with chronic diseases, including neurodegenerative conditions [7]. Additional strengths include the use of multiple blood-based biomarkers of AD, analyzing associations between adherence to dietary patterns and AD biomarkers across several levels of the biomarkers’ distribution, and accounting for genetic predisposition to dementia.

However, our study should be interpreted within the context of several limitations. First, the cross-sectional design and observational nature cannot exclude reverse causation nor residual confounding, although we adjusted the models for many sociodemographic, lifestyle, and morbidity variables. Second, the timing of dietary assessment in relation to the onset or progression of AD pathology may influence the observed associations, since dietary habits may change dramatically in older adults over the years due to numerous possible socio-economic (e.g., loneliness) and physiological changes (e.g., difficulties in chewing, swallowing, and reduced taste and smell acuity) [50,51]. Specifically, the effects of diet on AD pathology may take years to manifest and not be captured by cross-sectional studies. Third, despite using a validated FFQ, which is the most widely used tool to assess dietary intake in large cohorts, there are limitations to this method, causing possible dietary assessment biases. These include misreporting of certain foods (especially those perceived as less socially desirable) and inaccuracies in remembering food consumption over the specific period and portion size estimation [20]. Although dietary patterns offer several advantages over the study of single foods or nutrients, equal adherence scores can be obtained from substantially different combinations of their components, which in turn may have opposed associations with AD biomarkers. Fourth, changes in serum biomarker levels including Aβ 42 and Aβ 40 may be less pronounced than in CSF and not adequately reflect Aβ accumulation in the brain, as well as being hampered by peripheral production or alterations in their distribution and metabolism [52]. Furthermore, it is unclear how the levels of blood-based AD biomarkers are affected by physiological factors like circadian rhythm and food quantity and quality [53]. Fifth, blood biomarker levels can be altered by other present physical pathologies [17], potentially affecting eating habits as well. Specifically, diagnoses of several somatic diseases may be related to deterioration or improvements in diet quality [54], which suggests the possibility of reverse causality. Sixth, the study sample from SNAC-K comprised community dwelling, urban, mostly highly educated, relatively affluent Swedish older adults. Although we have no data on the ethnicity of participants, most of them were likely white. The absence of sufficient heterogeneity across these determinants, several of which are known risk factors for dementia [2,55], limits the generalizability of our findings to other populations and settings.

### 4.3 Conclusion

In this study, we showed associations between higher adherence to the MDS and lower levels of p-tau181, and between higher adherence to the EDII and higher concentrations of NfL at medium and/or high levels of said biomarkers. Higher adherence to the MDS was associated with an elevated Aβ42/40 ratio and lower NfL levels, but only at lower levels of the biomarkers. Certain associations between diet quality and AD biomarkers were only apparent among the oldest old (i.e., MDS and lower levels of GFAP) and stronger in *APOE-ε4* carriers (i.e., MDS and lower levels of p-tau181).

However, these results should be interpreted with caution because they rely on cross-sectional, observational data and cannot therefore prove causality. Future studies should use longitudinal designs and larger, more diverse samples. Our findings provide a deeper understanding of how dietary patterns are associated with AD pathology and suggest which individuals may benefit the most from adhering to these patterns. This can pave the way for designing precision public health interventions and providing personalized advice to older persons at risk for neurodegenerative diseases in the clinic.

## CRediT authorship contribution statement

**Anja Mrhar:** Writing – original draft, Visualization, Software, Methodology, Formal analysis. **Adrián Carballo-Casla:** Writing – review & editing, Visualization, Supervision, Software, Methodology, Formal analysis, Conceptualization. **Giulia Grande:** Writing – review & editing, Methodology. **Martina Valletta:** Writing – review & editing, Visualization, Software, Methodology. **Claudia Fredolini:** Writing – review & editing, Resources. **Laura Fratiglioni:** Writing – review & editing, Resources. **Milica Gregorič Kramberger:** Supervision, Resources, Project administration. **Aleš Kuhar:** Supervision, Resources. **Bengt Winblad:** Writing – review & editing, Resources, Project administration, Funding acquisition, Conceptualization. **Amaia Calderón-Larrañaga:** Writing – review & editing, Resources. **Davide Liborio Vetrano:** Writing – review & editing, Visualization, Supervision, Resources, Project administration, Methodology, Investigation, Funding acquisition, Conceptualization.

## Declaration of competing interest

The authors declare that they have no known competing financial interests or personal relationships that could have appeared to influence the work reported in this paper.

## Data Availability

Data are from the SNAC-K project, a population-based study on aging and dementia. Access to these original data is available to the research community upon approval by the SNAC-K organization. Applications for accessing these data can be submitted through http://www.snac-k.se/.

## Appendix Supplementary materials

Image, application 1
