# Supplementary material for: Dietary patterns and blood-based biomarkers of Alzheimer's disease in cognitively intact older adults: Findings from a population-based study
Source: J Prev Alzheimers Dis. 2025 Mar 14;12(6):100124. doi: 10.1016/j.tjpad.2025.100124 (PMC12434269; doi:10.1016/j.tjpad.2025.100124)
Supplement: Supplementary file 1 [file mmc1.docx]

# SUPPLEMENTARY MATERIAL

***Supplementary Table 1*** *Consumption of the components and scoring of the Mediterranean Diet Score (MDS)*

|  |  | **Total n=1907** | **Male n=755** | **Female n=1152** |
| --- | --- | --- | --- | --- |
|  | **Food components** | **Median (IQR)** | **Median (IQR)** | **Median (IQR)** |
| 1 | Vegetables (g/day) | 189.1 (137.4) | 155.3 (155.8) | 211.2 (145.8) |
| 2 | Legumes (g/day) | 6.4 (7.4) | 7.2 (8.0) | 5.9 (6.9) |
| 3 | Fruits and nuts (g/day) | 281 (195.7) | 245.4 (182.6) | 305.2 (200.3) |
| 4 | Cereals (g/day) | 242.4 (111.1) | 263.3 (116.0) | 228.7 (105.6) |
| 5 | Fish (g/day) | 47.6 (33.7) | 48.2 (35.0) | 47.2 (32.9) |
| 6 | Monounsaturated to saturated fat (ratio) | 1.03 (0.24) | 1.05 (0.24) | 1.02 (0.24) |
| 7 | Meat and poultry (g/day) | 86.4 (52.3) | 98.6 (59.2) | 78.5 (45.4) |
| 8 | Dairy (g/day) | 348.4 (23.9) | 318.8 (210.4) | 367.8 (243.0) |
| 9 | Alcohol (g/day) | 9.1 (9.0) | 12.2 (10.1) | 7.1 (7.6) |

IQR (interquartile range)

To calculate the MDS score, a value of 0 or 1 was assigned to each of the nine groups, with the sex-specific median as the cutoff. For the components (1) vegetables, (2) legumes, (3) fruits and nuts, (4) cereal, (7) fish, and (8) ratio of monounsaturated to saturated fatty acids, persons whose consumption was at or above the median were assigned a value of 1. For the components (7) meat and poultry and (8) dairy products, persons whose consumption was below the median were assigned a value of 1. For alcohol intake, a value of 1 was assigned to men who had between 10 and 50 g of ethanol per day and to women who consumed between 5 and 25 g per day. The total score range was from 0 to 9.

Vegetables = leafy or stem vegetables, root vegetables, bulbs, inflorescences and fruit vegetables, and other vegetables. Legumes = fresh and dried legumes. Fruits and nuts = fresh fruits and nuts and seeds. Cereals = grains and flours, pasta, bakery, breakfast cereals, and potatoes. Fish = white fish, oily fish, and seafood. Meat and poultry = pork, poultry, other meats (e.g., beef, lamb), offal, processed meat, and meat derivatives. Dairy = milk and milkshakes, yogurt and fermented milks, fresh cheese, mature cheese, and processed cheese.

**Supplementary Table 2** Consumption and inflammatory effect scores of the components of the Empirical Dietary Inflammatory Index.

|  | | **Total**  **N=1907** | **Overall inflammatory effect score** |
| --- | --- | --- | --- |
| ***Pro-inflammatory EDII components (portions/day)*** *^a^* | | **Mean (SD)** |  |
| 1 | Processed meat | 0.658 (0.699) | 165.03 |
| 2 | Red meat | 0.304 (0.234) | 140.19 |
| 3 | Organ meat | 0.033 (0.049) | 144.61 |
| 4 | Fish (other than dark-meat fish) | 0.224 (0.168) | 252.45 |
| 5 | Vegetables (other than dark yellow and leafy green) | 0.668 (0.613) | 136.14 |
| 6 | Refined grains | 1.385 (1.043) | 81.21 |
| 7 | High-energy beverages | 0.251 (0.525) | 156.85 |
| 8 | Low-energy beverages | (data not available) | 94.77 |
| 9 | Tomatoes | 0.664 (0.622) | 167.92 |
| ***Anti-inflammatory EDII components (portions/day)*** ^b^ | |  |  |
| 10 | Beer | 0.341 (.0509) | –136.99 |
| 11 | Wine | 0.322 (0.379) | –249.70 |
| 12 | Tea | 0.661 (0.824) | –42.25 |
| 13 | Coffee | 1.701 (1.081) | –83.18 |
| 14 | Dark yellow vegetables | 0.420 (0.488) | –165.37 |
| 15 | Leafy green vegetables | 0.588 (0.591) | –190.29 |
| 16 | Snacks | 0.140 (0.218) | –45.08 |
| 17 | Fruit juice | 0.375 (0.493) | –58.95 |
| 18 | Pizza | 0.022 (0.041) | –1175.21 |

The mean daily consumption of each food group by each participant was multiplied by the corresponding inflammatory effect score. The weighted consumption was summed to obtain the EDII and divided by 1000.

^a^ Wine = red or white wine. Coffee = soluble, espresso, Italian, or filtered coffee. Dark yellow vegetables = carrots, yellow (winter) squash, yams, or sweet potatoes. Leafy green vegetables = cooked or raw spinach, iceberg or head lettuce, or romaine or leaf lettuce. Snacks = potato chips, corn chips, popcorn, or crackers. Fruit juice = apple juice or cider, orange juice, grapefruit juice, or other fruit juice.

^b^ Processed meat = processed meats, bacon, or hot dog. Red meat = beef, pork, lamb, or hamburger patty. Organ meat = beef, calf, or pork liver, or chicken or turkey liver. Fish (other than dark-meat fish) = canned tuna, shrimp, lobster, scallops, fish, or other seafood other than dark-meat fish. Other vegetables = celery, mushrooms, green pepper, corn, mixed vegetables, eggplant, zucchini, alfalfa sprouts, or cucumber. Refined grains = white bread, English muffin, bagel or roll, muffin or biscuit, pancakes, or waffles; or white rice; or pasta. High-energy beverages = cola with sugar, other carbonated beverages with sugar, or fruit punch drinks. Low-energy beverages = low-energy cola or other low-energy carbonated beverages. Tomatoes = fresh tomato, tomato juice, or tomato sauce.

**Supplementary Table 3** Differences in associations of the Mediterranean Diet Score (MDS) and Empirical Dietary Inflammatory Index (EDII) (per 1-SD increment) with blood-based biomarkers of Alzheimer’s disease across levels of the biomarkers’ distributions.

|  | **MDS β (95% CI) ^a^** | | | **EDII β (95% CI) ^b^** | | |
| --- | --- | --- | --- | --- | --- | --- |
| **Biomarker percentiles** | 50^th^ vs 25^th^ | 75^th^ vs 25^th^ | 75^th^ vs 50^th^ | 50^th^ vs 25^th^ | 75^th^ vs 25^th^ | 75^th^ vs 50^th^ |
| **t-tau** |  |  |  |  |  |  |
| Model 3 | 0.022 [-0.021, 0.064] | 0.001 [-0.067, 0.068] | -0.021 [-0.080, 0.038] | 0.004 [-0.041, 0.033] | 0.024 [-0.030, 0.077] | 0.028 [-0.025, 0.081] |
| **p-tau181** |  |  |  |  |  |  |
| Model 3 | -0.027 [-0.051, -0.003]* | -0.032 [-0.066, -0.001]* | -0.005 [-0.034, 0.024] | -0.012 [-0.035, 0.011] | 0.002 [-0.037, 0.040] | 0.014 [-0.020, 0.048] |
| **Aβ 42/40** |  |  |  |  |  |  |
| Model 3 | -0.037 [-0.067, -0.006]* | -0.024 [-0.073, 0.024] | 0.012 [-0.026, 0.051] | -0.009 [-0.040, 0.021] | -0.014 [-0.065, 0.036] | -0.005 [-0.049, 0.039] |
| **NfL** |  |  |  |  |  |  |
| Model 3 | 0.006 [-0.006, 0.018] | 0.012 [-0.009, 0.034] | 0.006 [-0.013, 0.025] | 0.005 [-0.006, 0.016] | 0.023 [0.001, 0.045]* | 0.018 [-0.001, 0.038] |
| **GFAP** |  |  |  |  |  |  |
| Model 3 | -0.004 [-0.016, 0.007] | -0.003 [-0.022, 0.016] | 0.001 [-0.014, 0.017] | 0.005 [-0.006, 0.016] | 0.000 [-0.022, 0.023] | -0.005 [-0.024, 0.015] |

*p < 0.05; CI (confidence interval) ; Aβ40 = 40-aminoacid β amyloid peptide; Aβ42 = 42-aminoacid β amyloid peptide; t-Tau = total tau; p-Tau181 = phosphorylated tau 181; NfL = neurofilament light; GFAP = glial fibrillary acidic protein

Range of the dietary patterns: MDS: 0 to 9, 1-SD increment, 1.60; EDII: -1.106 to 2.773, 1-SD increment, 0.30

Range of the biomarkers (standardized): t-tau: -1.42 to 23.65; p-tau181: -1.03 to 15.45; Aβ 40 / Aβ 42: -2.36 to 23.04; NfL: 2.36 to 23.04; GFAP: -0.62 to 23.49

Biomarkers’ percentile values (standardized): t-tau (25^th^: -0.628, 50^th^: -0.193, 75^th^: 0.315); p-181 (25^th^: -0.544, 50^th^: -0.274, 75^th^: 0.116); Aβ 40/Aβ 42 (25^th^: -0.427, 50^th^: -0.095, 75^th^: 0.271); NfL (25^th^: -0.534, 50^th^: -0.353, 75^th^: -0.039), GFAP (25^th^: -0.375, 50^th^: -0.224, 75^th^: -0.012)

Bootstrapped quantile regression models adjusted as Model 3 in Table 2: sex, age, living arrangements (living alone or not), longest held occupation (manual worker or not), highest educational level (elementary school, high school, or university), smoking status (have never smoked, former smoker, current smoker, or no data), physical activity level [(i) sedentary: less than 0.5 hours/day of moderate physical activity, (ii) low active: ≥ 0.5 hours/day of moderate physical activity, (iii) active: ≥ 1 hour/day of moderate physical activity, (iv) very active: ≥ 3 hours/day of moderate physical activity or ≥ 1 hour/day of moderate and ≥ 1 hour/day of vigorous physical activity, or (v) no data], body mass index [<20, 20 to <25, 25 to <30, ≥30 kg/m^2^, or no data], energy intake (kcal/day), diabetes, heart diseases (atrial fibrillation, heart failure, ischemic heart disease, or heart valve disease), cerebrovascular disease, chronic lung disease (chronic obstructive pulmonary disease, emphysema, or chronic bronchitis), cancer (hematological and solid neoplasms), depression and mood diseases, hypertension, anemia, and chronic kidney disease.

**Supplementary Table 4** Sensitivity analyses. Associations between the Mediterranean diet score (MDS) and Empirical Dietary Inflammatory Index (EDII) (per 1-SD increment) and blood-based biomarkers of Alzheimer’s disease (at the 50^th^ percentile).

|  | **MDS β (95% CI)** | **EDII β (95% CI)** |
| --- | --- | --- |
| **t-tau** |  |  |
| Excluding MMSE <27 | 0.011 [-0.042, 0.064] | -0.017 [-0.060, 0.026] |
| Alternative version of the MDS | 0.022 [-0.024, 0.069] | Not applicable |
| Alternative version of the EDII 1 | Not applicable | 0.004 [-0.043, 0.051] |
| Alternative version of the EDII 2 | Not applicable | -0.015 [-0.067, 0.038] |
| **p-tau181** |  |  |
| Excluding MMSE <27 | -0.022 [-0.049, -0.002]* | 0.007 [-0.022, 0.036] |
| Alternative version of the MDS | -0.018 [-0.044, 0.007] | Not applicable |
| Alternative version of the EDII 1 | Not applicable | -0.005 [-0.029, 0.019] |
| Alternative version of the EDII 2 | Not applicable | -0.011 [-0.032, 0.010] |
| **Aβ 42/40** |  |  |
| Excluding MMSE <27 | -0.006 [-0.037, 0.025] | -0.006 [-0041, 0.030] |
| Alternative version of the MDS | 0.018 [-0.013, 0.050] | Not applicable |
| Alternative version of the EDII 1 | Not applicable | -0.014 [-0.045, 0.017] |
| Alternative version of the EDII 2 | Not applicable | -0.018 [-0.044, 0.008] |
| **NfL** |  |  |
| Excluding MMSE <27 | -0.015 [-0.029, -0.001]* | 0.010 [-0.004, 0.024] |
| Alternative version of the MDS | -0.003 [-0.015, 0.008] | Not applicable |
| Alternative version of the EDII 1 | Not applicable | 0.012 [-0.003, 0.028] |
| Alternative version of the EDII 2 | Not applicable | 0.004 [-0.008, 0.016] |
| **GFAP** |  |  |
| Excluding MMSE <27 | -0.005 [-0.017, 0.007] | 0.008 [-0.003, 0.018] |
| Alternative version of the MDS | -0.001 [-0.013, 0.010] | Not applicable |
| Alternative version of the EDII 1 | Not applicable | 0.004 [-0.011, 0.018] |
| Alternative version of the EDII 2 | Not applicable | 0.005 [-0.009, 0.018] |

*p < 0.05; CI = confidence interval; MMSE = Mini Mental State Examination; Aβ40 = 40-aminoacid β amyloid peptide; Aβ42 = 42-aminoacid β amyloid peptide; t-Tau = total tau; p-Tau181 = phosphorylated tau 181; NfL = neurofilament light; GFAP = glial fibrillary acidic protein

Alternative version of the MDS: scoring based on the sex-specific consumption of the MDS components in a Greek population

Alternative version of the EDII 1: pro-inflammatory scoring for snacks, beer, and pizza

Alternative version of the EDII 2: anti-inflammatory scoring for fish (other than dark-meat fish), other vegetables (i.e., vegetables other than leafy green vegetables and dark yellow vegetables), and tomatoes

Range of the dietary patterns: MDS: 0 to 9, 1-SD increment, 1.59; MDS (alternative version): 0 to 8, 1-SD increment, 1.16; EDII: -1.106 to 2.773, 1-SD increment, 0.30; EDII (alternative version 1): -0.810 to 3.160, 1-SD increment, 0.31; EDII (alternative version 2): -2.800 to 1.448, 1-SD increment, 0.40

Range of the biomarkers (standardized): t-tau: -1.42 to 23.65; p-tau181: -1.03 to 15.45; Aβ 40/Aβ 42: -2.36 to 23.04; NfL: 2.36 to 23.04; GFAP: -0.62 to 23.49

Biomarkers’ values at 50^th^ percentile (standardized): t-tau: -0.193; p-181: -0.274; Aβ 40/Aβ 42: -0.095; NfL: -0.353; GFAP: -0.224

Quantile regression models adjusted as Model 3 in Table 2: sex, age, living arrangements (living alone or not), longest held occupation (manual worker or not), highest educational level (elementary school, high school, or university), smoking status (have never smoked, former smoker, current smoker, or no data), physical activity level [(i) sedentary: less than 0.5 hours/day of moderate physical activity, (ii) low active: ≥ 0.5 hours/day of moderate physical activity, (iii) active: ≥ 1 hour/day of moderate physical activity, (iv) very active: ≥ 3 hours/day of moderate physical activity or ≥ 1 hour/day of moderate and ≥ 1 hour/day of vigorous physical activity, or (v) no data], body mass index [<20, 20 to <25, 25 to <30, ≥30 kg/m^2^, or no data], energy intake (kcal/day), diabetes, heart diseases (atrial fibrillation, heart failure, ischemic heart disease, or heart valve disease), cerebrovascular disease, chronic lung disease (chronic obstructive pulmonary disease, emphysema, or chronic bronchitis), cancer (hematological and solid neoplasms), depression and mood diseases, hypertension, anemia, and chronic kidney disease.

**Supplementary Table 5** *Associations between the Mediterranean Diet Score components (per 1-SD increment) and blood-based biomarkers of Alzheimer´s disease (at the 50th percentile).*

|  | | **t-tau** | **p-tau181** | **Aβ 40 / Aβ 42** | **NfL** | **GFAP** |
| --- | --- | --- | --- | --- | --- | --- |
| 1 | Vegetable | -0.019 (-0.068, 0.031) | -0.027 (-0.055, 0.001) | 0.019 (-0.011, 0.049) | 0.004 (-0.013, 0.020) | -0.009 (-0.024, 0.006) |
| 2 | Legumes | -0.017 (-0.026, 0.060) | -0.012 (-0.036, 0.012) | 0.017 (-0.015, 0.049) | -0.004 (-0.018, 0.009) | -0.006 (-0.025, 0.013) |
| 3 | Fruits and nuts | -0.012 (-0.052, 0.029) | -0.003 (-0.029, 0.032) | 0.015 (-0.017, 0.047) | 0.010 (-0.004, 0.024) | -0.012 (-0.025, 0.000) |
| 4 | Cereals | 0.032 (-0.026, 0.090) | -0.003 (-0.029, 0.031) | -0.028 (-0.068, 0.011) | -0.001 (-0.021, 0.019) | -0.010 (-0.028, 0.009) |
| 5 | Fish | 0.018 (-0.023, 0.059) | -0.003 (-0.029, 0.024) | -0.008 (-0.038, 0.021) | 0.003 (-0.010, 0.016) | 0.003 (-0.011, 0.017) |
| 6 | Monounsaturated fatty acids | -0.020 (-0.061, 0.021) | -0.008 (-0.027, 0.011) | 0.012 (-0.019, 0.043) | -0.006 (-0.022, 0.009) | -0.001 (-0.011, 0.009) |
| 7 | Meat | 0.036 (-0.020, 0.092) | 0.014 (-0.009, 0.037) | -0.049 (0.093, 0.005) | 0.002 (-0.014, 0.018) | 0.013 (-0.004, 0.030) |
| 8 | Dairy | 0.018 (-0.035, 0.072) | 0.019 (-0.010, 0.048) | -0.010 (-0.048, 0.027) | 0.000 (-0.015, 0.015) | -0.006 (-0.021, 0.009) |
| 9 | Alcohol | -0.021 (-0.074, 0.032) | -0.044 (-0.075, -0.014)*** | 0.043 (0.005, 0,081) | -0.013 (-0.028, 0.001) | -0.008 (-0.021, 0.006) |

***p < 0.05; CI (confidence interval) ; Aβ40 = 40-aminoacid β amyloid peptide; Aβ42 = 42-aminoacid β amyloid peptide; t-Tau = total tau; p-Tau181 = phosphorylated tau 181; NfL = neurofilament light; GFAP = glial fibrillary acidic protein

Range of the biomarkers (standardized): t-tau: -1.42 to 23.65; p-tau181: -1.03 to 15.45; Aβ 40 / Aβ 42: -2.36 to 23.04; NfL: 2.36 to 23.04; GFAP: -0.62 to 23.49

Biomarkers’ values at 50^th^ percentile (standardized): t-tau: -0.193; p-181: -0.274; Aβ 40/Aβ 42: -0.095; NfL: -0.353; GFAP: -0.224

Quantile regression models adjusted as Model 3 in Table 2: sex, age, living arrangements (living alone or not), longest held occupation (manual worker or not), highest educational level (elementary school, high school, or university), smoking status (have never smoked, former smoker, current smoker, or no data), physical activity level (sedentary, low active, active, very active, no data), body mass index (<20, 20 to <25, 25 to <30, ≥30 kg/m^2^, or no data), energy intake (kcal/day), diabetes, heart diseases (atrial fibrillation, heart failure, ischemic heart disease, or heart valve disease), cerebrovascular disease, chronic lung disease (chronic obstructive pulmonary disease, emphysema, or chronic bronchitis), cancer (hematological and solid neoplasms), depression and mood diseases, hypertension, anemia, and chronic kidney disease. Models were also adjusted for all other components of the Mediterranean Diet Score.

**Supplementary Table 6** *Association between the Empirical Dietary Inflammatory Index components (per 1-SD increment) and blood-based biomarkers of Alzheimer´s disease (at the 50th percentile).*

|  | | **t-tau** | **p-tau181** | **Aβ 40 / Aβ 42** | **NfL** | **GFAP** |
| --- | --- | --- | --- | --- | --- | --- |
| ***Pro-inflammatory EDII components*** | | | | | | |
| 1 | Processed meat | -0.039 (-0.081, 0.002) | -0.028 (-0.053, 0.002) | 0.022 (-0.012, 0.057) | 0.004 (-0.016, 0.024) | -0.002 (-0.017, 0.014) |
| 2 | Red meat | 0.034 (-0.018, 0.086) | 0.009 (-0.017, 0.035) | 0.007 (-0.038, 0.052) | 0.002 (-0.012, 0.016) | 0.009 (-0.008, 0.025 |
| 3 | Organ meat | -0.008 (-0.061, 0.046) | -0.001 (-0.025, 0.028) | 0.023 (-0.005, 0.050) | -0.004 (-0.020, 0.012) | 0.009 (-0.002, 0.021) |
| 4 | Fish (other than dark-meat fish) | -0.001 (-0.037, 0.034) | -0.010 (-0.034, 0.015) | 0.003 (-0.028, 0.034) | 0.001 (-0.016, 0.018) | -0.004 (-0.018, 0.011) |
| 5 | Vegetables (other than dark yellow and leafy green) | -0.041 (-0.100, 0.018) | 0.012 (-0.025, 0.049) | -0.027 (-0.063, 0.009) | 0.002 (-0.018, 0.022) | -0.000 (-0.017, 0.017) |
| 6 | Refined grains | -0.020 (-0.084, 0.044) | 0.007 (-0.025, 0.049) | -0.009 (-0.037, 0.020) | 0.011 (-0.009, 0.030) | 0.007 (-0.011, 0.025) |
| 7 | High-energy beverages | 0.002 (-0.053, 0.056) | 0.021 (-0.004, 0.046) | -0.012 (-0.045, 0.022) | 0.003 (-0.013, 0.020) | -0.005 (-0.020, 0.010) |
| 8 | Low-energy beverages | (data not available) | (data not available) | (data not available) | (data not available) | (data not available) |
| 9 | Tomatoes | 0.031 (-0.028, 0.090) | 0.019 (-0.009, 0.048) | -0.016 (-0.058, 0.026) | 0.013 (-0.008, 0.033) | 0.007 (-0.013, 0.027) |
| ***Anti-inflammatory EDII components*** | | | | | | |
| 10 | Beer | 0.025 (-0.013, 0.064) | -0.009 (-0.031, 0.041) | 0.005 (-0.022, 0.031) | -0.002 (-0.015, 0.011) | -0.002 (-0.013, 0.010) |
| 11 | Wine | -0.034 (-0.082, 0.013) | -0.036 (-0.059, -0.014)*** | 0.060 (0.027, 0.093) | -0.007 (-0.020, 0.007) | -0.002 (-0.016, 0.011) |
| 12 | Tea | -0.026 (-0.075, 0.027) | 0.009 (-0.017, 0.034) | -0.035 (-0.068, -0.002) | -0.009 (-0.022, 0.003) | -0.004 (-0.018, 0.009) |
| 13 | Coffee | -0.001 (-0.051, 0.049) | 0.003 (-0.019, 0.026) | 0.020 (-0.010, 0.050) | -0.004 (-0.018, 0.009) | -0.005 (-0.017, 0.007) |
| 14 | Dark yellow vegetables | -0.001 (-0.058, 0.056) | -0.015 (-0.041, 0.012) | 0.005 (-0.025, 0.035) | -0.002 (-0.019, 0.016) | -0.004 (-0.017, 0.009) |
| 15 | Leafy green vegetables | -0.005 (-0.050, 0.039) | -0.007 (-0.038, 0.023) | 0.014 (-0.031, 0.058) | -0.004 (-0.021, 0.013) | -0.005 (-0.022, 0.013) |
| 16 | Snacks | 0.004 (-0.042, 0.049) | 0.017 (-0.014, 0.048) | -0.012 (-0.041, 0.018) | 0.008 (-0.015, 0.032) | -0.006 (-0.020, 0.008) |
| 17 | Fruit juice | -0.015 (-0.062, 0.032) | -0.026 (-0,047, 0.001) | 0.010 (-0.018, 0.039) | -0.003 (-0.018, 0.011) | -0.004 (-0.015, 0.008) |
| 18 | Pizza | 0.004 (-0.036, 0.044) | -0.016 (0.045, 0.013) | 0.018 (-0.022, 0.059) | -0.004 (-0.021, 0.013) | -0.001 (-0.014, 0.012) |

***p < 0.05; CI (confidence interval) ; Aβ40 = 40-aminoacid β amyloid peptide; Aβ42 = 42-aminoacid β amyloid peptide; t-Tau = total tau; p-Tau181 = phosphorylated tau 181; NfL = neurofilament light; GFAP = glial fibrillary acidic protein

Range of the biomarkers (standardized): t-tau: -1.42 to 23.65; p-tau181: -1.03 to 15.45; Aβ 40/Aβ 42: -2.36 to 23.04; NfL: 2.36 to 23.04; GFAP: -0.62 to 23.49

Biomarkers’ values at 50^th^ percentile (standardized): t-tau: -0.193; p-181: -0.274; Aβ 40 / Aβ 42: -0.095; NfL: -0.353; GFAP: -0.224

Quantile regression models adjusted as Model 3 in Table 2: sex, age, living arrangements (living alone or not), longest held occupation (manual worker or not), highest educational level (elementary school, high school, or university), smoking status (have never smoked, former smoker, current smoker, or no data), physical activity level (sedentary, low active, active, very active, no data), body mass index (<20, 20 to <25, 25 to <30, ≥30 kg/m^2^, or no data), energy intake (kcal/day), diabetes, heart diseases (atrial fibrillation, heart failure, ischemic heart disease, or heart valve disease), cerebrovascular disease, chronic lung disease (chronic obstructive pulmonary disease, emphysema, or chronic bronchitis), cancer (hematological and solid neoplasms), depression and mood diseases, hypertension, anemia, and chronic kidney disease. Models were adjusted for all other components of the Empirical Dietary Inflammatory Index.

**Supplementary Table 7** *Association between the Alternative Mediterranean Diet (AMED) and the Dietary Inflammatory Index (DII) (per 1-SD increment) and blood-based biomarkers of Alzheimer´s disease (at the 25th, 50th, and 75th percentiles).*

|  | **AMED β (95% CI)** | | | **DII β (95% CI)** | | |
| --- | --- | --- | --- | --- | --- | --- |
| **Biomarker** | 25^th^ percentile | 50^th^ percentile | 75^th^ percentile | 25^th^ percentile | 50^th^ percentile | 75^th^ percentile |
| **t-tau** |  |  |  |  |  |  |
| Model 3 | -0.048 [-0.011, -0.084]* | 0.034 [-0.010, 0.078] | 0.013 [-0.056, 0.081] | 0.013 [-0.032, 0.058] | 0.014 [-0.052, 0.080] | 0.038 [-0.039, 0.115] |
| **p-tau181** |  |  |  |  |  |  |
| Model 3 | -0.026 [-0.006, -0.047]* | 0.009 [-0.016, 0.033] | 0.012 [-0.030, 0.053] | -0.011 [-0.042, 0.019] | -0.005 [-0.036, 0.025] | 0.005 [-0.041, 0.051] |
| **Aβ 42/40** |  |  |  |  |  |  |
| Model 3 | 0.035 [0.004, 0.066]* | 0.021 [-0.018, 0.060] | 0.002 [-0.054, 0.049] | -0.049 [-0.091, -0.006]* | -0.028 [-0.073, 0.016] | -0.041 [-0.100, 0.017] |
| **NfL** |  |  |  |  |  |  |
| Model 3 | -0.005 [-0.017, 0.008] | -0.002 [-0.015, 0.010] | 0.005 [-0.014, 0.025] | 0.004 [-0.015, 0.024] | 0.006 [-0.010, 0.021] | -0.001 [-0.032, 0.029] |
| **GFAP** |  |  |  |  |  |  |
| Model 3 | -0.004 [-0.015, 0.008] | -0.008 [-0.020, 0.003] | -0.012 [-0.032, 0.009] | 0.009 [-0.006, 0.024] | 0.006 [-0.011, 0.0.24] | 0.010 [-0.016, 0.037] |

*p < 0.05; CI (confidence interval) ; Aβ40 = 40-aminoacid β amyloid peptide; Aβ42 = 42-aminoacid β amyloid peptide; t-Tau = total tau; p-Tau181 = phosphorylated tau 181; NfL = neurofilament light; GFAP = glial fibrillary acidic protein

Range of the dietary patterns: AMED: 0 to 9, 1-SD increment, 1.68; DII: -5.06 to 5.43, 1-SD increment, 2.02

Range of the biomarkers (standardized): t-tau: -1.42 to 23.65; p-tau181: -1.03 to 15.45; Aβ 40 / Aβ 42: -2.36 to 23.04; NfL: 2.36 to 23.04; GFAP: -0.62 to 23.49

Biomarkers’ values at 50^th^ percentile (standardized): t-tau: -0.193; p-181: -0.274; Aβ 40/Aβ 42: -0.095; NfL: -0.353; GFAP: -0.224

Quantile regression models adjusted as Model 3 in Table 2: sex, age, living arrangements (living alone or not), longest held occupation (manual worker or not), highest educational level (elementary school, high school, or university), smoking status (have never smoked, former smoker, current smoker, or no data), physical activity level (sedentary, low active, active, very active, no data), body mass index (<20, 20 to <25, 25 to <30, ≥30 kg/m^2^, or no data), energy intake (kcal/day), diabetes, heart diseases (atrial fibrillation, heart failure, ischemic heart disease, or heart valve disease), cerebrovascular disease, chronic lung disease (chronic obstructive pulmonary disease, emphysema, or chronic bronchitis), cancer (hematological and solid neoplasms), depression and mood diseases, hypertension, anemia, and chronic kidney disease.

**Supplementary Table 8** *Association between the Dietary Approaches to Stop Hypertension (DASH) (per 1-SD increment) and blood-based biomarkers of Alzheimer´s disease (at the 25th, 50th, and 75th percentiles).*

|  | **DASH β (95% CI)** | | |
| --- | --- | --- | --- |
| **Biomarker** | 25^th^ percentile | 50^th^ percentile | 75^th^ percentile |
| **t-tau** |  |  |  |
| Model 3 | 0.040 [0.006, 0.073]* | 0.037 [-0.001, 0.075] | 0.038 [-0.014, 0.090] |
| **p-tau181** |  |  |  |
| Model 3 | -0.028 [-0.005, -0.051]* | -0.021 [-0.045, 0.004] | -0.045 [-0.009, -0.081]* |
| **Aβ 42/40** |  |  |  |
| Model 3 | 0.025 [-0.004, 0.055] | 0.005 [-0.026, 0.037] | -0.003 [-0.046, 0.040] |
| **NfL** |  |  |  |
| Model 3 | 0.005 [-0.005, 0.015] | 0.002 [-0.009, 0.014] | -0.000 [-0.024,0.024] |
| **GFAP** |  |  |  |
| Model 3 | -0.002 [-0.014, 0.010] | 0.000 [-0.013, 0.013] | -0.000 [-0.018, 0.018] |

*p < 0.05; CI (confidence interval) ; Aβ40 = 40-aminoacid β amyloid peptide; Aβ42 = 42-aminoacid β amyloid peptide; t-Tau = total tau; p-Tau181 = phosphorylated tau 181; NfL = neurofilament light; GFAP = glial fibrillary acidic protein

Range of the dietary pattern: 10 to 37, 1-SD increment, 4.29

Range of the biomarkers (standardized): t-tau: -1.42 to 23.65; p-tau181: -1.03 to 15.45; Aβ 40/Aβ 42: -2.36 to 23.04; NfL: 2.36 to 23.04; GFAP: -0.62 to 23.49

Biomarkers’ values at 50^th^ percentile (standardized): t-tau: -0.193; p-181: -0.274; Aβ 40 / Aβ 42: -0.095; NfL: -0.353; GFAP: -0.224

Quantile regression models adjusted as Model 3 in Table 2: sex, age, living arrangements (living alone or not), longest held occupation (manual worker or not), highest educational level (elementary school, high school, or university), smoking status (have never smoked, former smoker, current smoker, or no data), physical activity level (sedentary, low active, active, very active, no data), body mass index (<20, 20 to <25, 25 to <30, ≥30 kg/m^2^, or no data), energy intake (kcal/day), diabetes, heart diseases (atrial fibrillation, heart failure, ischemic heart disease, or heart valve disease), cerebrovascular disease, chronic lung disease (chronic obstructive pulmonary disease, emphysema, or chronic bronchitis), cancer (hematological and solid neoplasms), depression and mood diseases, hypertension, anemia, and chronic kidney disease.

**Supplementary Figure 1** Flow chart of the study participants.


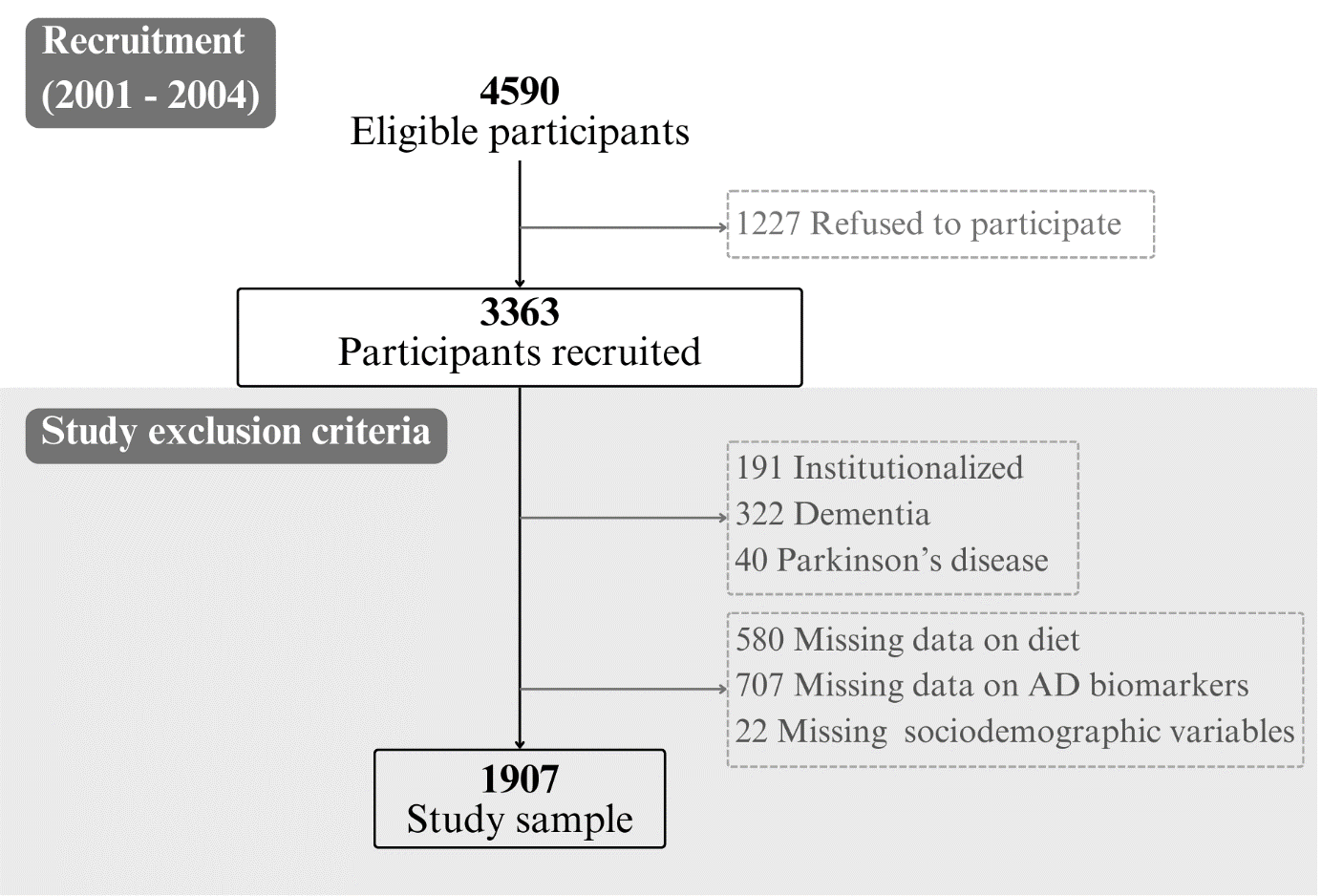


AD: Alzheimer’s disease

Note that one participant could fulfil more than one exclusion criterion

**Supplementary Figure 2** Directed acyclic graph (DAG) describing the potential causal and confounding effects of diet quality on biomarkers of Alzheimer’s disease.


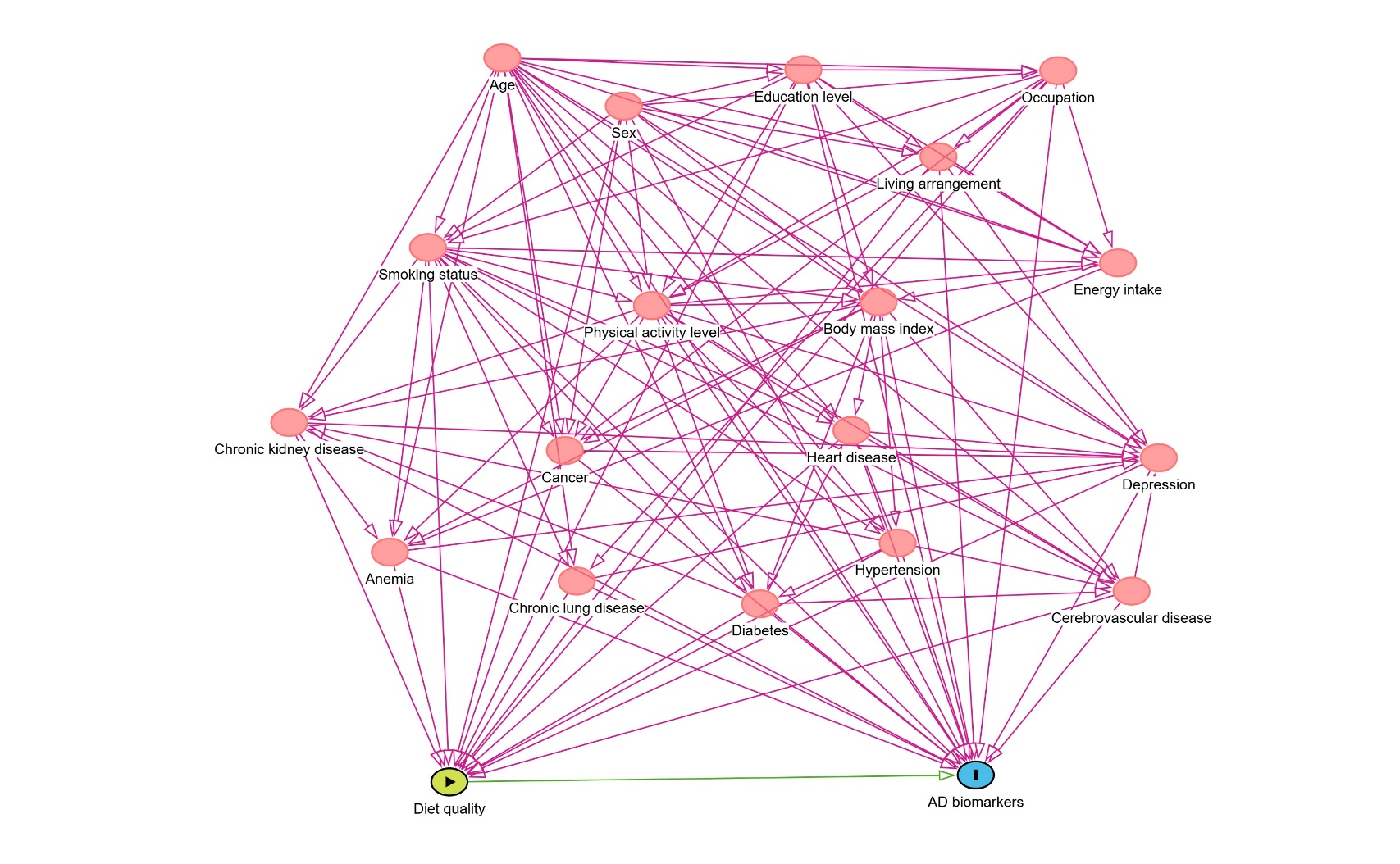


Green arrows indicate a causal path between the exposure and outcome of interest. Pink arrows indicate biasing paths (i.e., possible confounding factors modifying the association between the exposure and the outcome of interest).

**Supplementary Figure 3** Associations between the Mediterranean diet score (MDS) and Empirical Dietary Inflammatory Index (EDII) (per 1-SD increment) and blood-based biomarkers of Alzheimer’s disease (at the 25^th^ percentile).

Aβ40 = 40-aminoacid β amyloid peptide; Aβ42 = 42-aminoacid β amyloid peptide; t-Tau = total tau; p-Tau181 = phosphorylated tau 181; NfL = neurofilament light; GFAP = glial fibrillary acidic protein

Range of the dietary patterns: MDS: 0 to 9, 1-SD increment, 1.60; EDII: -1.106 to 2.773, 1-SD increment, 0.30. Range of the dietary patterns (standardized): MDS: 0 to 5.633; EDII: -3.711 to 9.304

Range of the biomarkers: t-tau: -1.42 to 23.65; p-tau181: -1.03 to 15.45; Aβ 40 / Aβ 42: -2.36 to 23.04; NfL: 2.36 to 23.04; GFAP: -0.62 to 23.49

Quantile regression models adjusted as Model 3 in Table 2: sex, age, longest held occupation (manual worker or not), highest educational level (elementary school, high school, or university), smoking status (have never smoked, former smoker, current smoker, or no data), physical activity level [(i) sedentary: less than 0.5 hours/day of moderate physical activity, (ii) low active: ≥ 0.5 hours/day of moderate physical activity, (iii) active: ≥ 1 hour/day of moderate physical activity, (iv) very active: ≥ 3 hours/day of moderate physical activity or ≥ 1 hour/day of moderate and ≥ 1 hour/day of vigorous physical activity, or (v) no data], body mass index [<20, 20 to <25, 25 to <30, ≥30 kg/m^2^, or no data], energy intake (kcal/day), diabetes, heart diseases (atrial fibrillation, heart failure, ischemic heart disease, or heart valve disease), cerebrovascular disease, chronic lung disease (chronic obstructive pulmonary disease, emphysema, or chronic bronchitis), cancer (hematological and solid neoplasms), depression and mood diseases, hypertension, anemia, and chronic kidney disease.

β (95% confidence intervals) were plotted for adherences to the dietary patterns above the 1^st^ percentile and below the 99^th^ percentile.

**Supplementary Figure 4** Associations between the Mediterranean diet score (MDS) and Empirical Dietary Inflammatory Index (EDII) (per 1-SD increment) and blood-based biomarkers of Alzheimer’s disease (at the 50^th^ percentile).

Aβ40 = 40-aminoacid β amyloid peptide; Aβ42 = 42-aminoacid β amyloid peptide; t-Tau = total tau; p-Tau181 = phosphorylated tau 181; NfL = neurofilament light; GFAP = glial fibrillary acidic protein

Range of the dietary patterns: MDS: 0 to 9, 1-SD increment, 1.60; EDII: -1.106 to 2.773, 1-SD increment, 0.30. Range of the dietary patterns (standardized): MDS: 0 to 5.633; EDII: -3.711 to 9.304

Range of the biomarkers: t-tau: -1.42 to 23.65; p-tau181: -1.03 to 15.45; Aβ 40 / Aβ 42: -2.36 to 23.04; NfL: 2.36 to 23.04; GFAP: -0.62 to 23.49

Quantile regression models adjusted as Model 3 in Table 2: sex, age, longest held occupation (manual worker or not), highest educational level (elementary school, high school, or university), smoking status (have never smoked, former smoker, current smoker, or no data), physical activity level [(i) sedentary: less than 0.5 hours/day of moderate physical activity, (ii) low active: ≥ 0.5 hours/day of moderate physical activity, (iii) active: ≥ 1 hour/day of moderate physical activity, (iv) very active: ≥ 3 hours/day of moderate physical activity or ≥ 1 hour/day of moderate and ≥ 1 hour/day of vigorous physical activity, or (v) no data], body mass index [<20, 20 to <25, 25 to <30, ≥30 kg/m^2^, or no data], energy intake (kcal/day), diabetes, heart diseases (atrial fibrillation, heart failure, ischemic heart disease, or heart valve disease), cerebrovascular disease, chronic lung disease (chronic obstructive pulmonary disease, emphysema, or chronic bronchitis), cancer (hematological and solid neoplasms), depression and mood diseases, hypertension, anemia, and chronic kidney disease.

β (95% confidence intervals) were plotted for adherences to the dietary patterns above the 1^st^ percentile and below the 99^th^ percentile.

**Supplementary Figure 5** Associations between the Mediterranean diet score (MDS) and Empirical Dietary Inflammatory Index (EDII) (per 1-SD increment) and blood-based biomarkers of Alzheimer’s disease (at the 75^th^ percentile).

Aβ40 = 40-aminoacid β amyloid peptide; Aβ42 = 42-aminoacid β amyloid peptide; t-Tau = total tau; p-Tau181 = phosphorylated tau 181; NfL = neurofilament light; GFAP = glial fibrillary acidic protein

Range of the dietary patterns: MDS: 0 to 9, 1-SD increment, 1.62; EDII: -1.106 to 2.773, 1-SD increment, 0.30. Range of the dietary patterns (standardized): MDS: 0 to 5.633; EDII: -3.711 to 9.304

Range of the biomarkers: t-tau: -1.42 to 23.65; p-tau181: -1.03 to 15.45; Aβ 40 / Aβ 42: -2.36 to 23.04; NfL: 2.36 to 23.04; GFAP: -0.62 to 23.49

Quantile regression models adjusted as Model 3 in Table 2: sex, age, longest held occupation (manual worker or not), highest educational level (elementary school, high school, or university), smoking status (have never smoked, former smoker, current smoker, or no data), physical activity level [(i) sedentary: less than 0.5 hours/day of moderate physical activity, (ii) low active: ≥ 0.5 hours/day of moderate physical activity, (iii) active: ≥ 1 hour/day of moderate physical activity, (iv) very active: ≥ 3 hours/day of moderate physical activity or ≥ 1 hour/day of moderate and ≥ 1 hour/day of vigorous physical activity, or (v) no data], body mass index [<20, 20 to <25, 25 to <30, ≥30 kg/m^2^, or no data], energy intake (kcal/day), diabetes, heart diseases (atrial fibrillation, heart failure, ischemic heart disease, or heart valve disease), cerebrovascular disease, chronic lung disease (chronic obstructive pulmonary disease, emphysema, or chronic bronchitis), cancer (hematological and solid neoplasms), depression and mood diseases, hypertension, anemia, and chronic kidney disease.

β (95% confidence intervals) were plotted for adherences to the dietary patterns above the 1^st^ percentile and below the 99^th^ percentile.
